# Chloroplast mini-barcodes combined with high resolution melting analysis to identify herbal medicine Difengpi (*Illicium difengpi*)

**DOI:** 10.1016/j.heliyon.2024.e38700

**Published:** 2024-09-27

**Authors:** Qian-Ru Zhou, Yun-Ying Ma, Hu-Qiang Lv, Zhao-Cen Lu, Li-Sheng Wang, Jun-Song Liang, Jing-Jian Li

**Affiliations:** a Guangxi Key Laboratory of Drug Discovery and Optimization, School of Pharmacy, Guilin Medical University, Guilin, 541004, China; b College of Pharmacy, Guilin Medical University, Guilin, 541004, China; c Xi'an Research Institute of Chinese Lacquer Under All China Federation of Supply and Marketing Cooperatives, Xi'an, 710061, China; d Guangxi Key Laboratory of Plant Conservation and Restoration Ecology in Karst Terrain, Guangxi Institute of Botany, Guangxi Zhuang Autonomous Region and Chinese Academy of Sciences, Guilin, 541006, Guangxi, China

**Keywords:** Herbal medicine safety, *Illicium difengpi*, Chloroplast genome, Mini-barcode, HRM assay, Species identification

## Abstract

Difengpi, derived from the air-dried stem bark of *Illicium difengpi* and enlisted in the Chinese Pharmacopoeia for its therapeutic effect against common ailments, confronts challenges due to dwindling wild resources and intentional substitution with potentially harmful botanical relatives. The imperative need to authenticate this herbal remedy has led to the development of robust methods. Here, we integrated chloroplast mini-barcoding and high resolution melting (HRM) analysis to distinguish Difengpi from the reported adulterants. We assembled the complete chloroplast (cp) genomes of *I. difengpi* and substituted close relatives *I. jiadifengpi* and *I. majus*, and conducted an in-depth comparative analysis to screen divergent regions for exploiting as DNA mini-barcodes. Despite the conservativeness characterizing the whole cp genomes among these *Illicium* species, we identified some highly variable regions with promising potential as molecular markers for species identification. Subsequently, we designed DNA mini-barcodes and subjected them to HRM analysis to assess their efficacy in species discrimination. Melting profiles unveiled that mini-barcodes designed from four divergence regions *trnL*–*trnF*, *trnF*–*ndhJ*, *ycf1*–*ndhF* and *rpl32*–*trnL* exhibited substantial discriminatory power, distinctly differentiated *I. difengpi* from *I. jiadifengpi*, *I. majus* and *I. verum*. We tested ten commercially available Difengpi products from online stores and local traditional markets using these four mini-barcodes. All ten samples clustered closely with the reference *I. difengpi* with genotype confidence higher than 93 %, indicating the presence of the claimed species in these samples, sans any reported toxic adulterants. Consequently, we simulated Difengpi mimic samples utilizing *I. jiadifengpi*, *I. majus* and *I*. *verum* and subjected them to evaluate the practicability of these mini-barcodes. The outcomes confirmed the precision of the four mini-barcodes in accurately discerning the mimic samples. In conclusion, integrating taxon-specific DNA mini-barcodes with HRM analysis is an efficient strategy for the authentication of species identity within commercial herbal products.

## Highlights

• Difengpi herbal medicine is beset with adulterants from toxic relatives.
• Chloroplast genome has potential in developing taxon-specific DNA mini-barcodes.
• Four mini-barcodes derived from cp genome could distinguish Difengpi from adulterants.
• DNA mini-barcode integrated with HRM is efficient in herbal medicine identification.

## Graphical abstract

**Figure:**
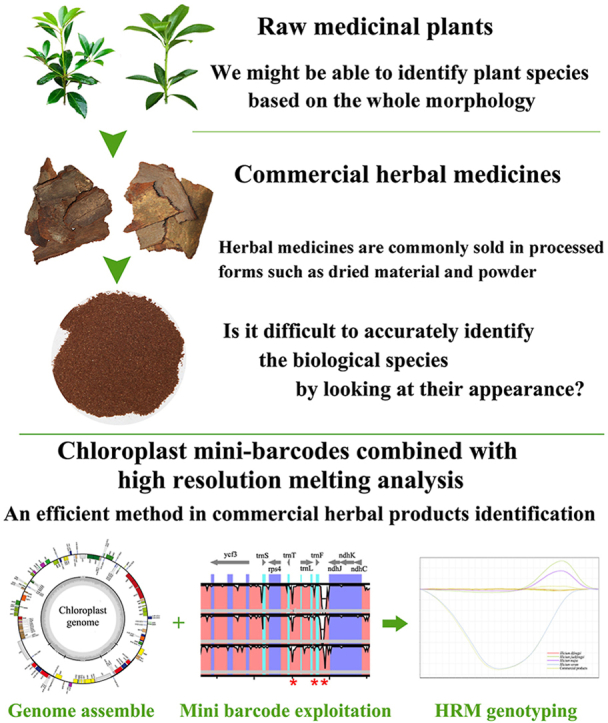

## 1 Introduction

The long-standing utilization of traditional herbal medicines, cherished as nature's bounty for healing diseases and preserving health, spans millennia. Despite the prevalence of chemically synthesized drugs today, herbal remedies still maintain their popularity in numerous countries, owing to their comparatively gentle efficacy resulting from the harmonious interplay of the internal constituents [1]. Notably, the intricate interactions among specific components can render herb distinct efficacy even among closely related species [2]. Consequently, substitutes and adulterants must be strictly prohibited to ensure herbs' therapeutic efficacy. However, in the past decades, the adulteration of herbal remedies has become a persistent issue. Unscrupulous merchants seek substantial profits by substituting rare herbal medicines with cheaper and less effective close relatives [[3], [4], [5]]. Aside from this deliberate fraud, the practitioner's inexact identification also leads to misuse and adulteration of the botanical remedies [6]. Whether it is deliberate practice or not, stringent quality control measures for herbal medicines become imperative to safeguard consumer health and ensure equitable trade practices.

**Fig. 1:**
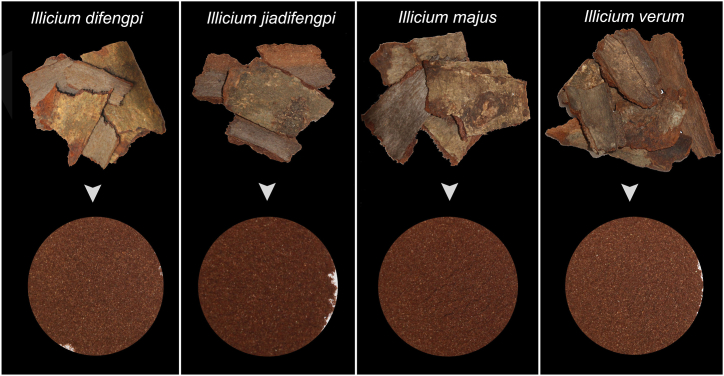
Commercial Difengpi material (*I. difengpi*) and adulterants (*I. jiadifengpi*, *I. majus* and *I*. *verum*) resemble each other.

Cortex herbal medicines encompass a category of medicinal materials derived from the stem bark or root bark of various medicinal plants. Among the extensive cortex medicinal materials, 19 find mention in the Chinese Pharmacopoeia as commonly utilized traditional herbal remedies. Difengpi is the famous one, derived from the dry stem bark of *Illicium difengpi* (Schisandraceae), an evergreen shrub distributed across the karst areas of Guangxi province, China [7]. Owing to its therapeutic efficacy in treating common diseases like rheumatic arthritis and lumbar muscle injury, this cortex herbal medicine has garnered attention recently [8]. However, the excessive collection of stem bark and its sluggish growth rate have placed the wild germplasm resources of *I. difengpi* at a perilous risk of extinction. Consequently, *I. difengpi* has been listed in the China Plant Red Data Book as a species under the category of Rare and Endangered Plants [7]. Simultaneously, the increasing demand for Difengpi in the Chinese herbal medicine trading market has catalyzed the emergence of adulterations. Previous investigations highlighted that adulterants of Difengpi primarily come from the barks of several closely related species within *Illicium* [8]. For example, the barks of *I. jiadifengpi* and *I. majus* are frequently found as substitutes in Difengpi products (Fig. 1). Some adverse reactions have arisen following the use of these adulterants, resulting in poisoning induced by toxic ingredients [9]. Particularly concerning are sesquiterpene lactones, such as anisatin found in *I. jiadifengpi* and *I. majus*, which are known to induce neurotoxicity (Huang et al., 2002; [10]). Moreover, the bark of *I. verum* is also occasionally substituted for Difengpi, but this fraud gains fewer attention due to its non-toxic nature. The processed barks of *I. difengpi*, *I. jiadifengpi*, *I. majus*, and *I. verum* show similar appearances, and consumer cannot tell them apart (Fig. 1). To avoid potential herb safety issues, precise authentication of species identity in herb products is necessary.

Various methods have been applied to identify cortex herbs, encompassing morphology and microstructure observation, chemical component analysis and DNA barcoding [6]. At the beginning, distinguishing Difengpi from counterfeits relied on scrutinizing color, texture, and microscopic features [11], but these methods heavily hinge on professional expertise and subjective perception, leading to divergent results. Chemical analyses assist in characterizing the essential composition; their results directly show the presence or absence of toxic compounds [12]. However, the accuracy of the result is affected by variances in bark materials collected at different growth stages, occasionally resulting in indistinguishable phytochemical profiles among closely related species [13]. Recently, DNA-based methods emerged as promising alternatives for plant species identification [4,14]. Notably, DNA barcoding gains prominence owing to its exceptional sensitivity, reproducibility, and straightforwardness. Unbound by morphological characteristics or physiological conditions, DNA barcoding offers species identification without specialized taxonomic expertise. This method uses universal primers to amplify standardized DNA barcodes, facilitating the establishment of a universal identification standard [5]. Numerous studies have consistently demonstrated the reliability of DNA barcoding in authenticating herbal medicine products [5,15]. Among them, an innovative approach combining DNA barcoding with high-resolution melting (HRM) analysis has gained attention for authenticating herbal medicines and quantifying adulterants in the products [16,17]. This method expedites herbal medicine identification, reducing labor and costs, and is highly amenable to high sample throughput owing to bypassing post-PCR treatments. However, current universal DNA barcodes face limitations in discriminating closely related species due to their low resolution within such taxa [18]. Moreover, complex processing of medicinal herb products lead to DNA degradation, rendering DNA barcoding ineffective when DNA fragments become smaller than universal barcodes. To overcome these challenges, DNA mini-barcodes, significantly reduced in size (≤250 bp), have proven effective in authenticating the species identity of processed foods and herbs [19,20]. Unlike universal DNA barcode, mini-barcode offers greater taxon specificity, enabling discrimination among closely related species [21]. Ideally, an effective DNA mini-barcode should contains sufficient variable sites within its reduced length. Exploiting the numerous divergent regions of chloroplast (cp) genome provides valuable sources for generating high-resolution mini-barcodes [14,22]. With the advent of next generation sequencing, exploiting such DNA mini-barcodes from whole cp genomes has become more feasible. Previously, we successfully developed species-specific DNA mini-barcodes from cp genomes, integrating them with HRM analysis to differentiate spice star anise from adulterants [14].

Here, we aimed to establish an integrated method for authenticating the species identity of Difengpi product. For this purpose, we assembled the cp genomes of *I. difengpi*, *I. jiadifengpi* and *I. majus*, and extracted the published cp genome of *I. verum* from NCBI. Through comprehensively comparison of these cp genomes, we found some hypervariable regions have potential in DNA mini-barcodes exploitation. Subsequently, twenty candidate mini-barcodes were evaluated through HRM analysis for species identification. As a result, four DNA mini-barcodes derived from *trnL*–*trnF*, *trnF*–*ndhJ*, *ycf1*–*ndhF* and *rpl32*–*trnL* exhibited exceptional power in distinguishing *I. difengpi* from *I. jiadifengpi*, *I. majus*, and *I. verum*. Furthermore, genotyping of commercial Difengpi products proved these four DNA mini-barcodes adept in identifying processed herbal products, affirming their robustness and efficacy.

## 2 Materials and methods

### 2.1 Sample collection and DNA extraction

Fresh leaves of *I. difengpi* and *I. verum* were obtained from Guangxi Institute of Botany, China, while *I. jiadifengpi* and *I. majus* were collected from wild resources and has been verified by botanist. Corresponding voucher specimens (voucher number: Illicium20210812001, Illicium20210812002, and Illicium20210812003) have been archived in the Herbarium of College of Pharmacy, Guilin Medical University. The lab-made Difengpi mimic samples were formulated using substituted species (i.e., *I. jiadifengpi*, *I. majus* and *I. verum*) according to the processing described in the Chinese Pharmacopoeia. In detail, the stem-bark of *I. jiadifengpi*, *I. majus* and *I. verum* were peel off and dried up in the sun for 72 h, and then these dried barks were broken into small pieces. Ten commercial Difengpi products were randomly acquired from herb vendors, including eight shops in Yulin Yinfeng International Chinese Medicine Port and two in other cities that offer online sales (Table S1). Genomic DNA extraction of fresh leaves from the four target species was accomplished utilizing the Plant Genomic DNA Kit (Tiangen Biotech Co., China). Commercial and mimic materials accumulated abundant secondary metabolites, especially polyphenols, so a specialized pretreatment was conducted prior to DNA extraction [14]. The pulverization of samples using liquid nitrogen to prevent DNA further degradation, followed by successive washes utilizing a cold solution comprising 1.4 mmol/L sodium chloride, 3 % polyvinylpyrrolidone (PVP), 20 mmol/L ethylene diamine tetraacetic acid and 100 mmol/L Tris-HCl, and repeated wash three times. These chemical reagents were purchased from Sangon Biotechnology Co. Ltd. (Shanghai, China). After this treatment, the genomic DNA was extracted using the aforementioned plant genomic DNA kit (Tiangen Biotech Co., China). The purity and concentration of DNA was assessed using agarose gel electrophoresis and NanoDrop 2000 (Thermo Scientific, United States), respectively.

### 2.2 Chloroplast genome sequencing and assembly

The DNA libraries with an insert size of 300 bp were constructed for *I. difengpi*, *I. jiadifengpi*, and *I. majus*. Subsequent sequencing was performed utilizing the Illumina HiSeq 2000 platform (Illumina, United States), generating paired-end reads with an average length of 150 bp, totaling 10 Gb of raw data for each species. Raw reads were filtered to obtain high-quality reads using the NGS QC Toolkit v2.3.3 to eliminate adapter tags and low-quality reads. Given abundant nuclear and mitochondrial DNA reads within the libraries, we used a reference-assisted method to filter cp putative reads prior to genome assembly. We mapped the clean reads to the previously published cp genome of *I. verum* (NC_034689) using Bowtie2 program [23]. The mapped reads were considered as the putative cp DNA sequences. We used SPAdes software to *de novo* assemble the putative cp reads into contigs by linking the overlapping regions [24]. Contigs supported by mate-pair information were merged into a coherent, extended scaffold. Considering the significant impact of parameter settings on assembly outcomes, particularly the K-mer parameter [25], a systematic assembly was conducted by setting K-mer = 21, 55, 85, and 115 in single-end mode, followed by using a Perl script to calculate the N50 of assembly results under different K-mer parameters to identify the best assembly model. The assembly sequence exhibiting the highest N50 was retained as the draft cp genome. In case the draft genome contained any gaps, we mapped all the reads back to it to fill them.

### 2.3 Genome annotation and divergence hotspot identification

The cp genome was annotated through the online annotation tool GeSeq [26] with *I*. *verum* (NC_034689) as the reference. Predictions for all tRNAs were carried out using tRNAscan-SE v2.0.7. To ensure annotation accuracy, the fasta format file of cp genome was also submitted to CPGAVAS2 [27], facilitating the generation of sequence files of genes, particularly the protein-coding genes. All genes underwent manual verification and correction by comparison with homologous genes from *I*. *verum*, especially for genes containing notably small exon (e.g., petB, petD, rpl16) and RNA editing start codon (e.g., ndhD, rps19, cemA). The circular cp genomes of *I. difengpi*, *I. jiadifengpi*, and *I. majus* were visualized using the OGDRAW tool [28]. A comparative analysis of the cp genomes among the four *Illicium* species was performed using the mVISTA program in Shuffle-LAGAN mode [29]. The divergent hotspots were visualized with *I. difengpi* as the reference. Furthermore, sequences from the divergent hotspots were extracted for alignment to find conserved sequences flanking the divergent loci for mini-barcode design.

### 2.4 Mini-barcode exploitation and high resolution melting analysis

Generally, a DNA mini-barcode with robust identification efficiency should encompass adequate variable loci while maintaining a minimal length. Adhering to this criterion, we sought suitable conserved sites at the flank of variable loci for primer design (Fig. S1). Subsequently, the efficacy of all primer pairs and the discriminatory capacity of mini-barcodes were rigorously evaluated through HRM analysis. Each 20 μL HRM reaction mixture comprised 20 ng genomic DNA of the tested *Illicium* plants, 1x HRM mix (Tiangen Biotech Co., China), and 0.5 μL of 5 mM forward and reverse primers. This HRM mix incorporates EvaGreen saturation dye renowned for its high-resolution properties, making it compatible with challenging DNA samples. Owing to its non-inhibitory nature, EvaGreen saturates the binding capacity of double-stranded PCR products even at high concentrations, enabling discrimination of single-base differences [30]. The HRM operation was done on a Rotor Gene Q (QIAGEN, Germany) real-time PCR instrument using a 36-well rotor, following a program that commenced with a 3 min pre-denaturation at 95 °C, 40 cycles comprising 20 s each of denaturation at 95 °C, annealing at 50–55 °C, and extension at 72 °C. Fluorescence data acquisition was performed at the “HRM” channel when each cycle finished. HRM profile generation involved a temperature ramp from 62 °C to 80 °C, ascending by 0.1 °C per step, each followed by a 2-s interval. To optimize genotyping resolution, we just kept the divergent melt phase by adjusting the starting and ending fluorescent signal. The melting curves can be visualized as either a normalized melting plot or a difference plot. Different species can be separated according to the curve shifting in the normalized melting plot. Meanwhile, difference plots were also used to aid visual interpretation since it could maximize the difference between *I. difengpi* and close relatives. In difference plot, when *I. difengpi* designated as the reference, its melting curve was rendered as a horizontal line. Under this condition, if the sequence of close relative contains any variable loci, their melting curves will deviate from that of *I. difengpi*. Here, the threshold of genotype confidence percentage were set as 90 % for genotyping these species. If the genotype confidence percentage of a melting curve is below 90 %, the corresponding sample is considered a different species.

### 2.5 HRM combined with mini-barcode to identify difengpi products

To assess the viability of cp mini-barcode coupled with HRM assay in authenticating commercial products, ten samples claimed as “Difengpi” were procured from various retail shops were tested. Unfortunately, even if we used the above mentioned washing solution to treat the samples, the DNA quality cannot meet the requirement of HRM assay. To address this problem, a specialized pre-amplification was conducted prior to HRM assay. Initially, the DNA of commercial samples were amplified using mini-barcode primers. PCR amplification was conducted in a 10 μL reaction mixture comprising 2 μL DNA template, 1x Taq PCR mix (Sangon Biotech Co., China), and 0.5 μL of 5 mM forward and reverse primers. Notably, we cannot use those PCR mix containing loading dye, since it potentially affect the successful execution of HRM reactions. PCR reaction involved denaturation at 95 °C for 3 min, followed by 30 cycles (94 °C for 20 s, 50–52 °C for 15 s, and 72 °C for 15 s). Subsequently, the PCR product was diluted to 20 ng/μL and utilized as a template for the HRM reaction. For genotyping species identity in the commercial samples, authenticated plant species' reference DNA was concurrently processed with the commercial samples in the same round HRM reaction. Clustering of the melting curve of a commercial sample with that of an authenticated plant species, exhibiting a genotype confidence percentage exceeding 90 %, classified them as the same species.

## 3 Results

### 3.1 Chloroplast genome features of four *Illicium* species

**Fig. 2:**
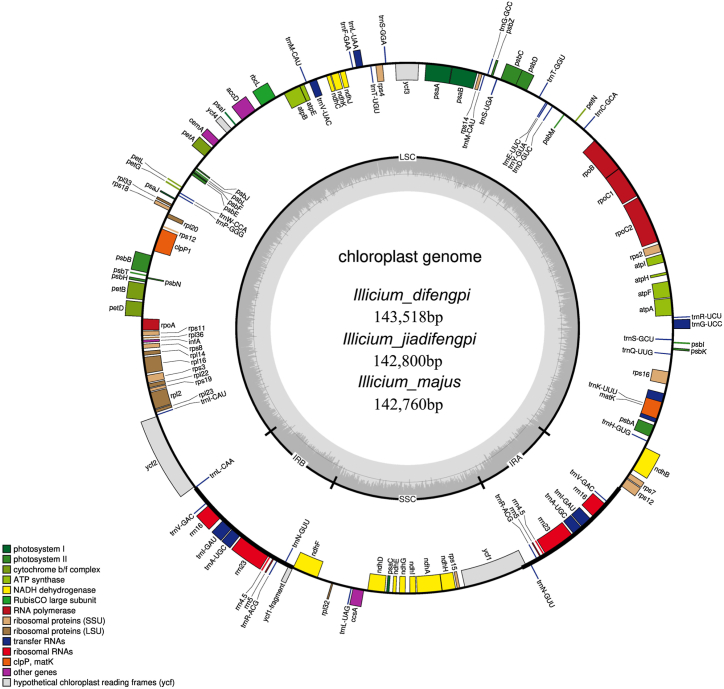
Structure map of the three *Illicium* chloroplast genomes. Genes shown on the outside of the circle are transcribed clockwise, and genes inside are transcribed counter-clockwise. The dark gray inner circle corresponds to the GC content, the light-gray to the AT content.

In this investigation, DNA libraries featuring an insert size of 300 bp were constructed. Illumina 150bp paired-end sequencing yielded distinct clean reads for each *Illicium* species: 27,779,214 for *I. difengpi*, 20,465,949 for *I. jiadifengpi*, and 25,586,144 for *I. majus*. Subsequent assembly of the cp genome was accomplished by aligning these clean reads to the reference cp genome of *I. verum*, yielding a moderate amount of cp putative reads for each species: 1,818,751 for *I. difengpi*, 6,210,242 for *I. jiadifengpi*, and 3,593,521 for *I. majus*. These reads provided a coverage of 35.4x, 30.9x, and 93.4 x for the cp genome of *I. difengpi*, *I. jiadifengpi*, and *I. majus*, suggesting the relatively large genome size within *Illicium* species, and distinct genome size among these species. The length of cp genome was 142,760 bp for *I. majus*, 142,800 bp for *I. jiadifengpi*, and 143,518 bp for *I. difengpi*, respectively (Fig. 2, Table 1). They exhibiting a typical quadripartite structure encompassing a large single copy (LSC), a small single copy (SSC), and short inverted repeat regions (IRa and IRb) flanking the SSC (Fig. 2). All three assembled cp genomes have been deposited in GenBank under the accession numbers OL802928 (*I. difengpi*), OL802930 (*I. jiadifengpi*), and OL802932 (*I. majus*). Remarkably, these cp genomes share an identical set of 113 unique genes, including 79 protein-coding, 30 tRNA, and 4 rRNA genes (Table 1). Gene distribution revealed 67 protein-coding genes and 24 tRNA genes residing in the LSC region, while 12 protein-coding genes and one tRNA are situated in the SSC region. Within the IR regions, 5 tRNA and 4 rRNA genes are duplicated, while no protein-coding genes in these regions. The gene content of *I. difengpi*, *I. jiadifengpi*, and *I. majus* were consistent with that of *I*. *verum* (Table 1). We also found a consistent GC content across these cp genomes, 39.1 % for *I. difengpi*, *I. majus*, and *I*. *verum*, and 39.2 % for *I. majus* (Table 1). Examination of IR borders revealed an identical boundaries in LSC/IRa, LSC/IRb, and SSC/IRa regions across all four species. These findings collectively illustrated a high degree of conservation within the cp genome of *Illicium* species.

**Table 1:** Comparison of chloroplast genome contents of *I*. *difengpi* and close relatives.

Characteristics	*I. difengpi*	*I. jiadifengpi*	*I. majus*	*I. verum*
Total size (bp)	143,518	142,800	142,760	143,187
LSC length (bp)	101,396	100,927	100,833	100,867
SSC length (bp)	20,274	20,133	20,081	20,230
IR length (bp)	10,924	10,870	10,923	11,045
GC content (%)	39.1	39.2	39.1	39.1
Total genes	113	113	113	113
Protein genes	79	79	79	79
tRNA	30	30	30	30
rRNA	4	4	4	4

### 3.2 Identification of divergent hotspots

**Fig. 3:**
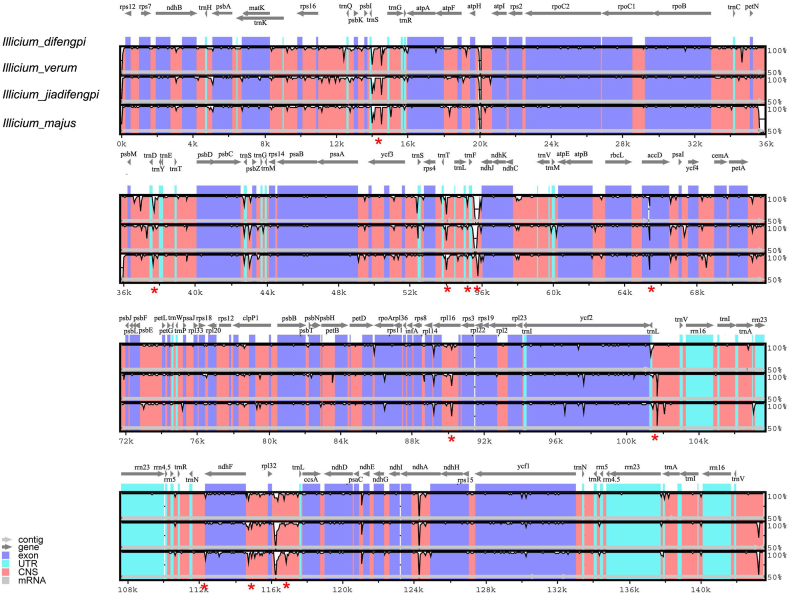
Sequence divergence plots in the four *Illicium* chloroplast genomes, using *I. difengpi* as reference. Arrows indicate the annotated genes and their transcriptional direction. The y-axis represents the percent identity within 50–100 %. The divergent hotspot candidates are marked by red stars. (For interpretation of the references to color in this figure legend, the reader is referred to the Web version of this article.)

To screen potential mini-barcode candidates for species identification, we aligned and visually assessed the complete cp genomes of the four *Illicium* species utilizing mVISTA, with the annotated cp genome of *I. difengpi* serving as the reference (Fig. 3). The analysis revealed a significant synteny and sequence similarity among these species, particularly within the IR regions. Despite the higher conservation observed in cp genomes, several sequence divergences were discerned in intergenic regions. Specifically, high sequence variations were noted in *trnS*–*trnG*, *trnD*–*trnY*, *trnT*–*trnL*, *trnL*–*trnF*, *trnF*–*ndhJ*, *accD*, *rpl16*, *trnL*–*trnV*, *trnN*–*ndhF*, *ndhF*–*rpl32*, *rpl32*–*trnL* (Fig. 3). These divergent regions exhibited nucleotide diversity values ranging from 0.0047 (*trnN*–*ndhF*) to 0.0201 (*trnF*–*ndhJ*) among the four *Illicium* species, indicating their potential as sources for mini-barcode exploitation. A good DNA mini-barcode should contain adequate variable loci while maintaining a minimal length. We prioritized the conserved sequences near the variable loci for primer design. With this aim, we designed 20 candidate barcodes from these divergent regions, generating amplicons ranging from 90 to 540 bp (Table S2). Although mini-barcodes shorter than 250 bp are recommended, designing such length barcodes for some divergent regions is challenging because no suitable primer sites were found near the variable loci. For example, the short and suitable barcode for *trnF*–*ndhJ* could only be designed with a length span of 390 bp. Considering these divergent regions exhibited high nucleotide variation and might be helpful to resolve the issue of species identification for Difengpi, we retained the barcodes derived from these regions even though their length was beyond the recommended range.

### 3.3 Finding applicable mini-barcodes for discrimination among four *Illicium* species

**Fig. 4:**
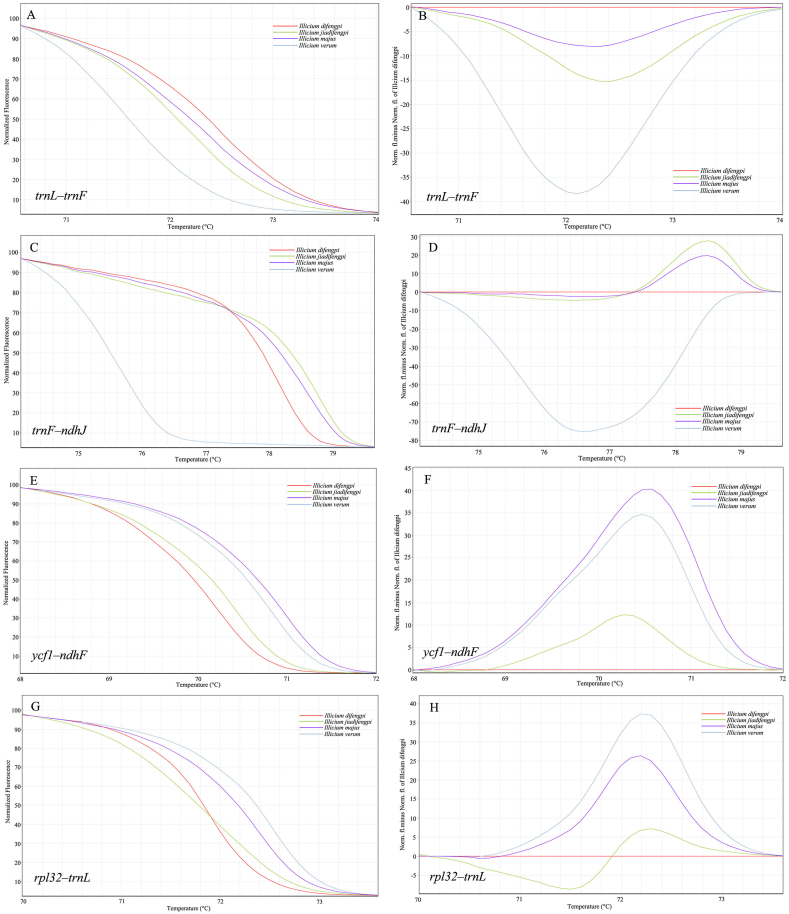
Melting curves of four *Illicium* species generated by HRM using *trnL*–*trnF*, *trnF*–*ndhJ*, *ycf1*–*ndhF*, and *rpl32*–*trnL*, respectively. (A, C, E, G) The melting curves shown on normalized plots. (B, D, F, H) The melting curves shown on difference plots.

We employed HRM to assess the discriminatory efficacy of these newly designed mini-barcodes across the four *Illicium* species, scrutinizing their melting profiles and genotype confidence percentage. Meanwhile, we should note that the melting temperature depends on both the length and the GC content of the mini-barcode. In some cases, despite nucleotide variations detected within some mini-barcodes, they did not impact the number of hydrogen bonds (e.g., guanine-cytosine (G) to cytosine (C) transversions), resulting in unaltered melting temperatures across these species. Based on their discriminatory performance, the 20 mini-barcodes were categorized into four grades (Fig. 4 and Figs. S2–S17): Incapable of differentiating any of the four *Illicium* species from one another. Capable of distinguishing *I. difengpi* from one adulterated species. Capable of distinguishing *I. difengpi* from two adulterated species. Capable of distinguishing *I. difengpi* from three adulterated species. Here, we aim to develop the last grade mini-barcode that could at least discern *I. difengpi* from its adulterated counterparts, irrespective of distinguishing between the adulterated species themselves. Following rigorous screening, four mini-barcodes, *trnL*–*trnF*, *trnF*–*ndhJ*, *ycf1*–*ndhF* and *rpl32*–*trnL*, met this grade (Table 2). The analysis of normalized melt curves using these mini-barcodes revealed that they distinctly differentiated *I. difengpi* from *I. jiadifengpi*, *I. majus* and *I*. *verum* (Fig. 4A, C, 4E and 4G). In difference plot, when *I. difengpi* was designated as the reference genotype, its melting curve was turn into a horizontal line, while other species exhibited distinct melting curve shapes (Fig. 4B 4D, 4F and 4H). Using a genotype confidence percentage threshold of 90 %, *I. jiadifengpi*, *I. majus* and *I*. *verum* were clearly designated as distinct genotypes. Furthermore, by setting each species as the reference genotype alternately and with a threshold of 0.00 % for genotype confidence, we could calculate the genotype similarity among the four species. In the *trnF*–*ndhJ* barcode, the highest genotype confidence (53.89 %) was observed between *I. jiadifengpi* and *I. majus*, whereas the lowest (0.00 %) was between *I. verum* and the other three species (Table 3). For *trnL*–*trnF* mini-barcode, the highest genotype confidence (55.83 %) was identified for *I. jiadifengpi* vs. *I. majus*, while the lowest genotype confidence was observed in *I. verum* vs. *I. difengpi*, and *I. verum* vs. *I. majus* (Supplemental Table S4). In *ycf1*–*ndhF* mini-barcode, *I. verum* vs. *I. majus* had the highest genotype confidence of 55.03 %, whereas the lowest (0.00 %) was between *I. difengpi* and *I. majus*, *I. difengpi* and *I. verum*, *I. jiadifengpi* and *I. majus* (Supplemental Table S5). In *rpl32*–*trnL* mini-barcode, the highest genotype confidence (45.10 %) was observed between *I. difengpi* and *I. jiadifengpi*, whereas the lowest (0.00 %) was between *I. verum* and *I. difengpi* (Supplemental Table S6). According to the genotype confidence given above, we concluded that all these four mini-barcodes not only could distinguish *I. difengpi* from *I. jiadifengpi*, *I. majus*, and *I. verum*, but also could distinguish *I. jiadifengpi*, *I. majus* and *I. verum* from each other.

**Table 2:** Characteristics of mini barcodes from four divergent regions.

Mini-barcode	Primer sequence (5′→3′)	Tm (°C)	Expected size (bp)
*trnL–trnF*	AGCCAAATCCTTGTTTTCTGA	52	90
	TTGAGTCTCTGCACCTATCCTT		
*trnF–ndhJ*	ACGGATTTCTCTATCTAGATGG	50	390
	GGATGAGCAAAGCCAATAG		
*ycf1–ndhF*	CAGCATTCAATGTGTATTCCTG	51	150
	CGAATCTCTTCTTACCTATTCTTG		
*rpl32–trnL*	GTTTTTCCCATCGATTCACT	50	150
	ATCGTTCATACTTGTTGCAGA

**Table 3:** Genotype confidence percentage of *trnF–ndhJ* between four *Illicium* species.

Species	*I. difengpi*	*I. jiadifengpi*	*I. majus*	*I. verum*
*I. difengpi*	100.00	0.17	4.66	0.00
*I. jiadifengpi*	0.17	100.00	53.89	0.00
*I. majus*	4.66	53.89	100.00	0.00
*I. verum*	0.00	0.00	0.00	100.00

### 3.4 HRM analysis of mini-barcode genotyping difengpi products

**Fig. 5:**
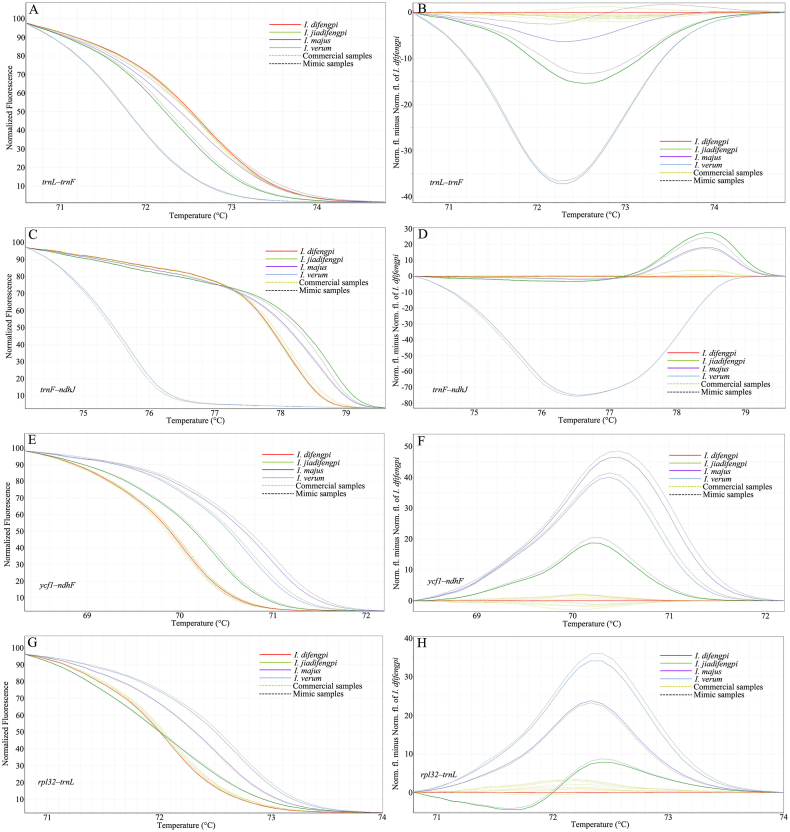
HRM genotyping the commercial and mimic Difengpi samples using *trnL*–*trnF*, *trnF*–*ndhJ*, *ycf1*–*ndhF*, and *rpl32*–*trnL*. (A, C, E, G) The melting curves shown on normalized plots. (B, D, F, H) The melting curves shown on difference plots. Solid lines with different color represent the authenticated plants. Yellow dotted lines represent commercial samples from market, while black dotted lines represent mimic samples made in our lab. (For interpretation of the references to color in this figure legend, the reader is referred to the Web version of this article.)

In our efforts to authenticate commercial Difengpi products using the mini-barcode combined with HRM analysis, DNA extraction from ten samples was undertaken, followed by PCR-HRM to profile the melting curves of *trnL*–*trnF*, *trnF*–*ndhJ*, *ycf1*–*ndhF* and *rpl32*–*trnL*, respectively. A key improvement in DNA quality was observed when samples were treated with a washing solution before extraction. Some heavily processed samples did not yield DNA without this treatment, while others' extracted DNA displayed a brownish hue (Fig. S18), indicating poor quality. Notably, samples treated with the washing solution could yield a slightly yellow hue DNA (Fig. S18), but still, with a deficient concentration, failed to reach the HRM requirement. A feeble fluorescence signal was obtained when we applied this DNA for HRM genotyping. To further improve this situation, *trnL*–*trnF*, *trnF*–*ndhJ*, *ycf1*–*ndhF* and *rpl32*–*trnL* fragment were amplified using Taq DNA mix with reaction of 30 cycles, and the PCR products were adjusted to 20 ng/μL for PCR-HRM analysis. By employing *I. difengpi* melting curve as reference genotype and setting the genotype confidence threshold of 90 %, automatic genotyping were performed for all Difengpi samples. The results revealed that ten samples exhibited similar melting curve shapes consistent with *I. difengpi*, displaying a genotype confidence percentage exceeding 93 % in the four mini-barcodes, confirming their identity as *I. difengpi* (Fig. 5). Given that no toxic adulterants were found in these commercial products, we simulated Difengpi mimic samples utilizing *I. jiadifengpi*, *I. majus* and *I*. *verum* and subjected them to evaluate mini-barcodes' discrimination efficiency. The outcomes also confirmed the precision of the four mini-barcodes in accurately discerning the mimic samples (Fig. 5). Furthermore, we tried to use traditional ITS2 barcode to authenticate these commercial samples and mimic samples but failed to generate ITS2 PCR amplicons, indicating a severe DNA degradation in these samples.

## 4 Discussion

In this study, HRM integrated with DNA mini-barcode was successfully developed to authenticate species identity in Difengpi products using newly designed cp mini-barcodes. DNA barcoding has been extensively utilized in many fields, with ITS2 being proposed as a pivotal barcode for the identification of herbal medicine [5]. However, traditional DNA barcoding is unsuitable for identifying Difengpi products because significant DNA degradation results in unsuccessful PCR amplification. This phenomenon is not unique to Difengpi, as most herbal materials sold on the market have been processed via cooking, drying, grinding, and alkali modification, leading to substantial DNA fragmentation [19]. If these damaged DNA fragments are smaller than traditional barcodes like ITS, *psbA*–*trnH*, *trnL*–*trnF*, *matK* and *rbcL*, they cannot be used for traditional DNA barcoding. Despite ITS2 having an approximate length of 250 bp, the PCR product's span using universal ITS2 primers exceeds 600 bp [14]. This notable length disparity highlights the potential benefit of species-specific DNA mini-barcodes in overcoming degradation challenges. As mini-barcodes typically focus on resolving specific taxon phylogenetic relationships, they can be designed to maintain high species discriminatory abilities and robust PCR amplification success rates [22]. Mini-barcodes can be obtained from nuclear or plastid genomes or traditional barcodes. Considering cost-effectiveness, sequencing and searching the whole cp genome to exploit mini-barcodes is the primary choice. Recent studies have successfully extracted mini-barcodes from fast-evolving loci within the cp genome to identify processed herb materials [19].

The *Illicium* genus poses taxonomic challenges due to the relatively slow evolvement and morphology that resemble each other. Consequently, understanding phylogenetic relationships at the species level in *Illicium* has yet to be addressed [31]. To discover efficient DNA mini-barcodes for distinguishing Difengpi from closely related adulterants, sequencing the cp genomes of these species becomes imperative. Thus, we assembled the cp genomes of *I. difengpi* and its adulterated species *I. jiadifengpi* and *I. majus* (Fig. 2). Comparative analysis revealed identical gene content and order among *I. difengpi*, *I. jiadifengpi*, *I. majus* and *I*. *verum*, with striking similarity in protein-coding sequence (Fig. 3). Despite the common use of *rbcL*, *matK*, *psbA*–*trnH* in inferring species evolutionary relationships, we observed minimal sequence variation in these barcodes among four *Illicium* species. These outcomes supported the conservative nature of *Illicium* cp genomes, akin to observations in monocotyledons of *Acorus* and *Oryza* [32]. While inter-species divergence within protein-coding genes is rare, some divergent sequences were detected in several intergenic spacers, including *trnS*–*trnG*, *trnD*–*trnY*, *trnT*–*trnL*, *trnL*–*trnF*, *trnF*–*ndhJ*, *trnL*–*trnV*, *trnN*–*ndhF*, *ndhF*–*rpl32* and *rpl32*–*trnL* (Fig. 3). Among them, *trnL*–*trnF* is a traditional DNA barcode widely used for plant taxonomy and evolution. Besides, the rapidly evolving loci *trnS*–*trnG*, *ndhF*–*rpl32* and *rpl32*–*trnL* are also prevalently found in other angiosperms plants and have been employed for inferring phylogenetic relationships under specific taxa and authenticating species identity [[33], [34], [35]]. Together, the divergent regions found in *Illicium* cp genomes might also have potential in distinguishing these closely related species.

Unlike traditional DNA barcoding, we utilized HRM analysis to validate the species discrimination power of newly designed mini-barcodes. HRM analysis genotyping species through melting behaviour of sequence variants within the barcode. This approach bypasses DNA sequencing and hence saves both time and cost. We assessed the melting curve shapes and genotype confidence percentages to screen which mini-barcodes could differentiate *I. difengpi* from *I. jiadifengpi*, *I. majus* and *I*. *verum*. Among the 20 mini-barcodes examined, *trnL*–*trnF*, *trnF*–*ndhJ*, *ycf1*–*ndhF* and *rpl32*–*trnL* effectively differentiated *I. difengpi* from other species (Fig. 4). In contract, the rest of the barcodes displayed either aberrant amplification plots or undistinguishable melting plots between *I. difengpi* and close relatives. Multiple factors such as poor primers, nonspecific products, and unusual sequence motifs can make abnormal HRM results difficult to interpret. In *Illicium* cp genome, we observed abundant poly structure flanking the hypervariable loci, making it almost impossible to design good primers. Considering these divergent regions exhibited high nucleotide variation and might be helpful for inferring phylogenetic relationships within *Illicium*, we recommended exploiting these divergent regions as long DNA barcodes instead of mini-barcodes for traditional DNA barcoding.

The shortest length mini-barcode with the same variable loci is considered the best for a given divergent region. Larger barcode can be analyzed successfully, but excess consistent sequence would lower discrimination. That is why the guideline for HRM analysis suggests that the amplicon is no larger than 250 bp. However, if a barcode contains enough variable loci, its length can be extended appropriately, such as the barcode *trnF*–*ndhJ* with a length of 390 bp still efficiently distinguishes *I. difengpi* from three close relatives (Fig. 5). Likewise, traditional DNA barcodes *psbA*–*trnH* and ITS2 combined with HRM have effectively identified species in *Artemisia* and *Sideritis* [36,37]. Therefore, regardless of DNA degradation, those barcodes longer than 250 bp but with high species discrimination power can be used as an alternative for HRM analysis. Besides DNA degradation, another challenge in HRM genotyping herbal products is the accumulated oxidants in the processed materials. We found that poor HRM reaction is also caused by oxidants, leading to inconclusive or low resolution. Previously, various modified extraction methods were proposed for extracting good quality DNA from different forms of herbal materials [6,38,39]. In these proposed methods, polyphenols are frequently mentioned oxidants that severely affect DNA quality, and polyvinylpyrrolidone is an effective polyphenol-removing reagent. Accordingly, we pretreated the Difengpi powder using a specially formulated buffer to remove oxidants before DNA extraction. Even though this treatment helps to improve DNA quality, DNA quantity is still insufficient for PCR-HRM analysis. This result is not a surprise because the Difengpi sample is derived from the old bark, which consists of outside dead tissue periderm and inside tissue cortex with abundant cellulose, lignin and polyphenols, and most of the dead cells have lost the nucleus [40]. Fortunately, DNA quantity is enough for standard PCR amplification, allowing us to amplify the target fragment for normal PCR-HRM reaction.

## 5 Conclusion

We identified some divergent regions for designing taxon-specific DNA mini-barcodes based on the cp genome data. In combination with HRM analysis, *trnL*–*trnF*, *trnF*–*ndhJ*, *ycf1*–*ndhF*, and *rpl32*–*trnL* mini-barcodes were found efficient in genotyping *I. difengpi* and its close relatives. Regarding the adulteration identification of Difengpi products, no adulterants were found in the collected samples, but we could not conclude this result for all Difengpi products throughout the markets. Consequently, we simulated Difengpi mimic samples utilizing *I. jiadifengpi*, *I. majus* and *I*. *verum* to confirm the practicability of four newly designed mini-barcodes. Overall, this integrated strategy is worth reference for the identification of Difengpi herbal medicine.

## CRediT authorship contribution statement

**Qian-Ru Zhou:** Formal analysis, Methodology, Writing – original draft. **Yun-Ying Ma:** Formal analysis, Methodology. **Hu-Qiang Lv:** Formal analysis, Resources. **Zhao-Cen Lu:** Resources. **Li-Sheng Wang:** Formal analysis, Investigation. **Jun-Song Liang:** Funding acquisition, Supervision. **Jing-Jian Li:** Funding acquisition, Project administration, Supervision, Writing – review & editing.

## Declaration of competing interest

The authors declare that they have no known competing financial interests or personal relationships that could have appeared to influence the work reported in this paper.
