# Supplementary material for: Chloroplast mini-barcodes combined with high resolution melting analysis to identify herbal medicine Difengpi (Illicium difengpi)
Source: Heliyon. 2024 Sep 27;10(19):e38700. doi: 10.1016/j.heliyon.2024.e38700 (PMC11471493; doi:10.1016/j.heliyon.2024.e38700)
Supplement: Multimedia component 1 [file mmc1.docx]

**Table S1. Detailed information of the commercial samples**

| Product label | Declared plant source | Medicinal part | Collecting locality | Sample ID |
| --- | --- | --- | --- | --- |
| Difengpi | *I. difengpi* | Bark | Yulin, Guangxi | COM_01 |
| Difengpi | *I. difengpi* | Bark | Yulin, Guangxi | COM_02 |
| Difengpi | *I. difengpi* | Bark | Yulin, Guangxi | COM_03 |
| Difengpi | *I. difengpi* | Bark | Yulin, Guangxi | COM_04 |
| Difengpi | *I. difengpi* | Bark | Yulin, Guangxi | COM_05 |
| Difengpi | *I. difengpi* | Bark | Yulin, Guangxi | COM_06 |
| Difengpi | *I. difengpi* | Bark | Yulin, Guangxi | COM_07 |
| Difengpi | *I. difengpi* | Bark | Yulin, Guangxi | COM_08 |
| Difengpi | *I. difengpi* | Bark | Taobao (Bozhou) | COM_09 |
| Difengpi | *I. difengpi* | Bark | Taobao (Baoding) | COM_10 |

**Table S2. Detail of 20 mini-barcodes for HRM analysis**

| Primer name | Primer sequence (5′→3′) | Divergence region | Tm (°C) | Amplicon size  (bp) |
| --- | --- | --- | --- | --- |
| I.DFP01_F | CGAAAGAAATATCTCCGATAGC | *trnS-trnG* | 52 | 90 |
| I.DFP01_R | GCCAGTACCTAGCCAGGC |  |  |  |
| I.DFP02_F | CAATTGCATGAATAGTGGAGG | *trnS-trnG* | 54 | 110 |
| I.DFP02_R | GTTGTGCAAGAATCCATAGCT |  |  |  |
| I.DFP03_F | ATGGGACCGCTGCAATAG | *psbM-trnD* | 55 | 150 |
| I.DFP03_R | CCAATCCAAGGAAGAGGATAC |  |  |  |
| I.DFP04_F | GAAATGAGGTGTACAGCCCAC | *trnD-trnY* | 55 | 310 |
| I.DFP04_R | TCCACATGTTCTGATGTTGCT |  |  |  |
| I.DFP05_F | CGGATCTTCGCTATGAATATGA | *trnT-trnL* | 54 | 320 |
| I.DFP05_R | CGTAATACTGGAACGGTCGAT |  |  |  |
| I.DFP06_F | AGCCAAATCCTTGTTTTCTGA | *trnL-trnF* | 52 | 90 |
| I.DFP06_R | TTGAGTCTCTGCACCTATCCTT |  |  |  |
| I.DFP07_F | ACGGATTTCTCTATCTAGATGG | *trnF-ndhJ* | 52 | 390 |
| I.DFP07_R | GGATGAGCAAAGCCAATAG |  |  |  |
| I.DFP08_F | TGGGGAATCAAAATGAAGC | *trnF-ndhJ* | 53 | 80 |
| I.DFP08_R | GAGTGAGAGGACTCTCATTCCT |  |  |  |
| I.DFP09_F | GACTGTCAAGAGTCAATTTGGT | *atpB–rbcL* | 53 | 120 |
| I.DFP09_R | GCGCAACCCAATCCTTGT |  |  |  |
| I.DFP10_F | GTAATGGTGACAGTTATTCCGT | *accD* | 52 | 170 |
| I.DFP10_R | AGTTGCGATTGTAACTCTTAGG |  |  |  |
| I.DFP11_F | CCGAGTCAATCTCCTCAGTCTA | *rpl16* | 53 | 120 |
| I.DFP11_R | TAGATGAATCCGGTTCATAGGA |  |  |  |
| I.DFP12_F | CATTTCACCATCAAGGCAT | *ycf2-trnL* | 51 | 95 |
| I.DFP12_R | GATCGAGAAAGATCTCTTGTTC |  |  |  |
| I.DFP13_F | CCCATAGGTTTGATCCTGTAGA | *trnL-trnV* | 55 | 540 |
| I.DFP13_R | CCAACAGTTCATCACGGAAG |  |  |  |
| I.DFP14_F | CAGCATTCAATGTGTATTCCTG | *ycf1-ndhF* | 51 | 150 |
| I.DFP14_R | CGAATCTCTTCTTACCTATTCTTG |  |  |  |
| I.DFP15_F | GCATAGTCATATGTACGTTCCA | *ndhF-rpl32* | 51 | 240 |
| I.DFP15_R | AGAAATACGTGGGAAAGCAT |  |  |  |
| I.DFP16_F | CTCGTCATTTCCCATAGAACTA | *ndhF-rpl32* | 52 | 110 |
| I.DFP16_R | CTAGTCGCCAATCTGTTAAATG |  |  |  |
| I.DFP17_F | AATTCTTCTGTCTACTGGTTCC | *rpl32-trnL* | 51 | 190 |
| I.DFP17_R | AAAACGACAATTCCCATCTC |  |  |  |
| I.DFP18_F | GTTTTTCCCATCGATTCACT | *rpl32-trnL* | 50 | 150 |
| I.DFP18_R | ATCGTTCATACTTGTTGCAGA |  |  |  |
| I.DFP19_F | CTGCAACAAGTATGAACGATG | *rpl32-trnL* | 52 | 85 |
| I.DFP19_R | CATATCTGATATCTGTATGGGACA |  |  |  |
| I.DFP20_F | GGAGTGAAGGAAAATCCACTAG | *rpl32-trnL* | 50 | 210 |
| I.DFP20_R | CCAGGAACGTTTATTAATACAA |  |  |  |

Note: Some intergenic region contain more than one divergent loci, and thus we design primers for each locus separately. The four mini-barcodes have capacity for genotyping Difengpi products are highlighted in blue.

In the following, we taking *trnF-ndhJ* for example to design HRM primer:


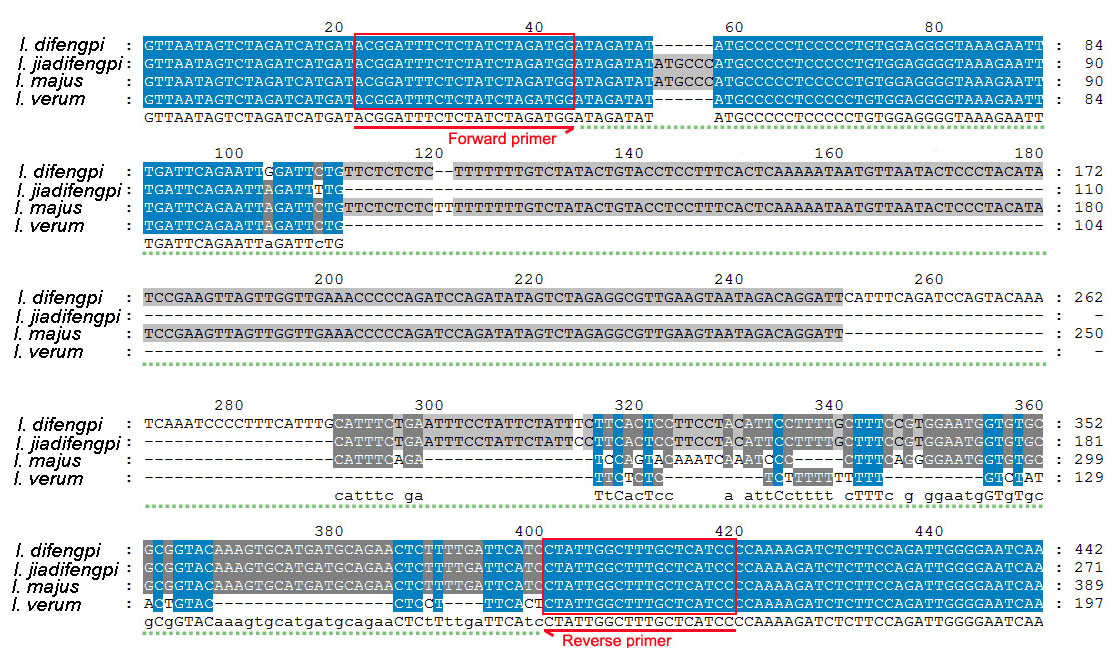


**Figure S1. HRM primer design**

The taxonomic resolution of a DNA barcode is affected by both divergent and conservative sequence. The more conservative sequence contain, the lower taxonomic resolution in HRM melting profile. Accordingly, those conserved sequence sites at the flank of divergent loci are given priority to design primer. As the schematic diagram shown above, those conserved sequence in the red box are designed as primers for *trnF-ndhJ* barcode (highlighted with green dotted line). Although the sequences located at 60-100bp are conserved and consistent among these species, they are not suitable for designing primers.

The following melting curves are the results of remaining HRM primers grouped into four types:


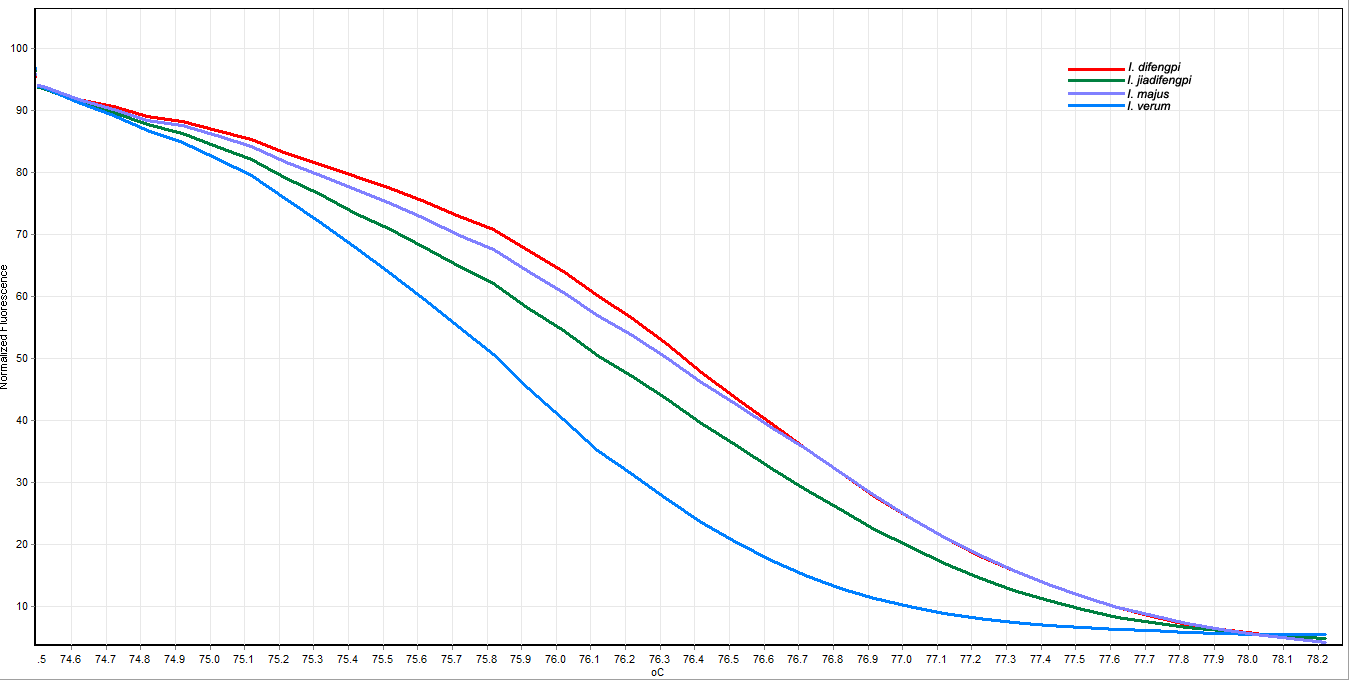


**Figure S2. Melting curves generated by primers I.DFP01_F/I.DFP01_R.** This primer pair cannot differentiate *I. difengpi* from *I. majus*.


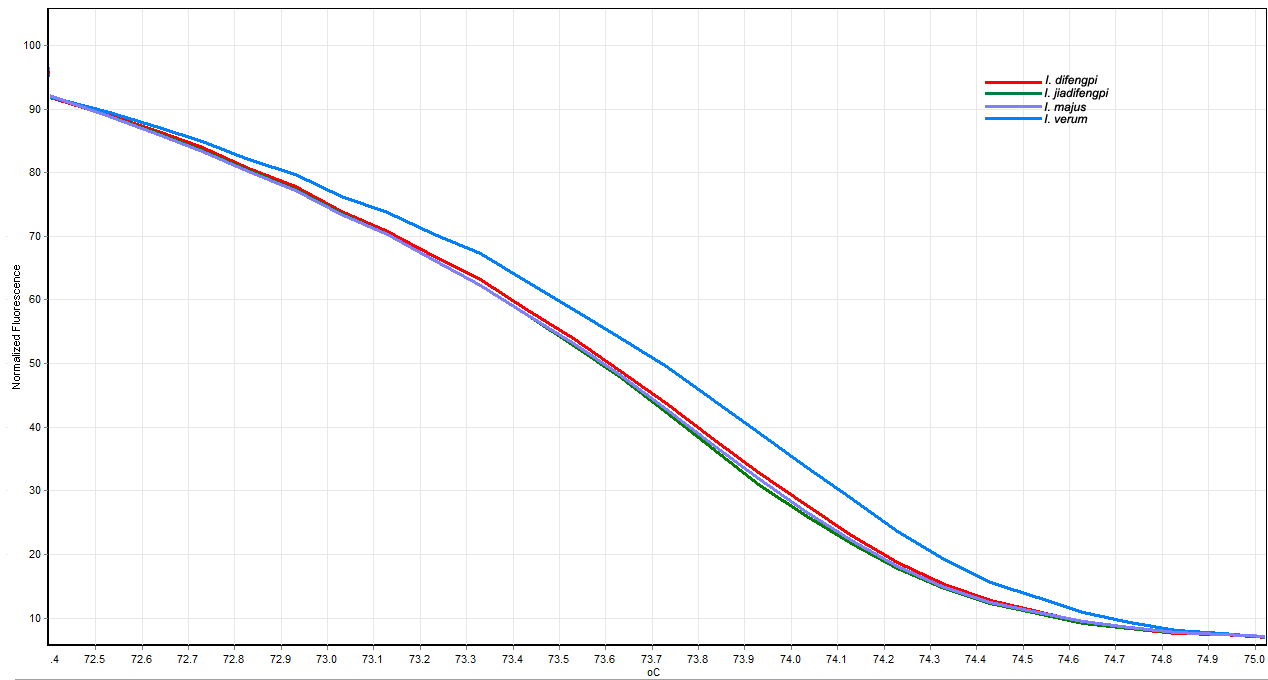


**Figure S3. Melting curves generated by primers I.DFP02_F/I.DFP02_R.** This primer pair cannot differentiate *I. difengpi*, *I. majus*, *I. jiadifengpi* and *I. verum* from each other.


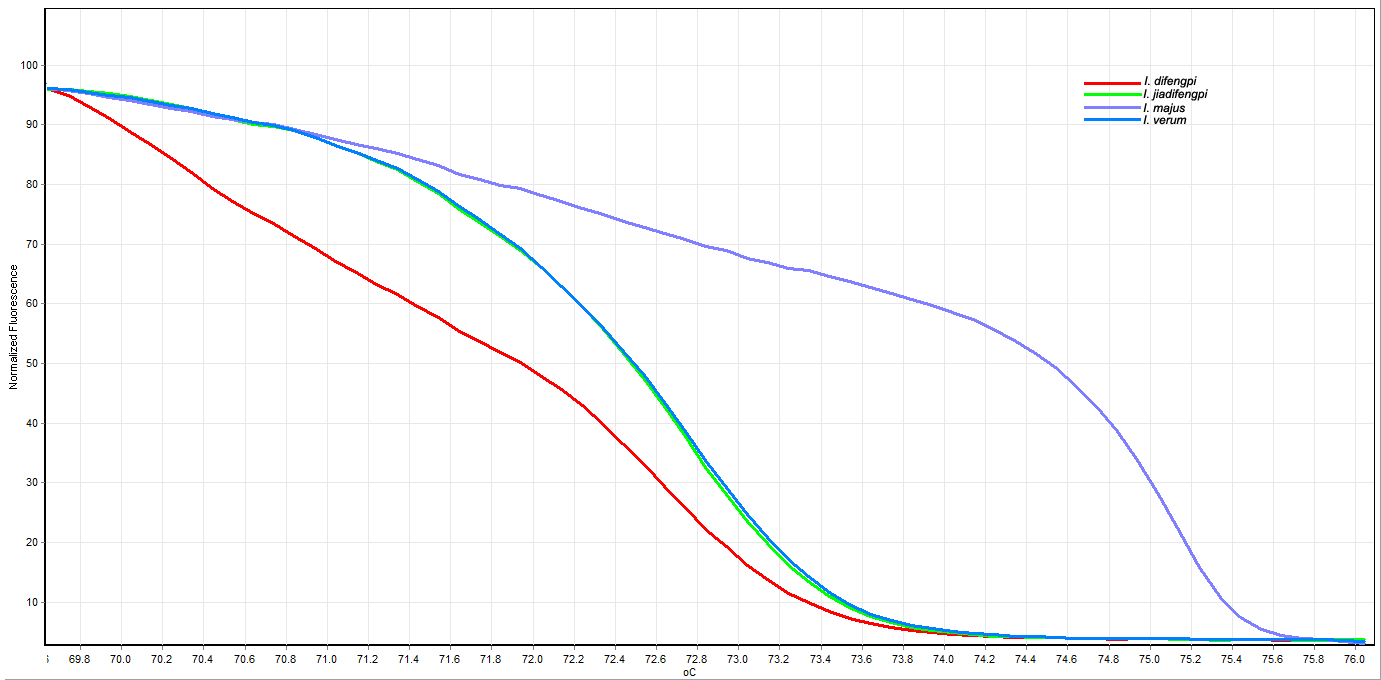


**Figure S4. Melting curves generated by primers I.DFP03_F/I.DFP03_R.** This primer pair cannot differentiate *I. verum* from *I. jiadifengpi*.


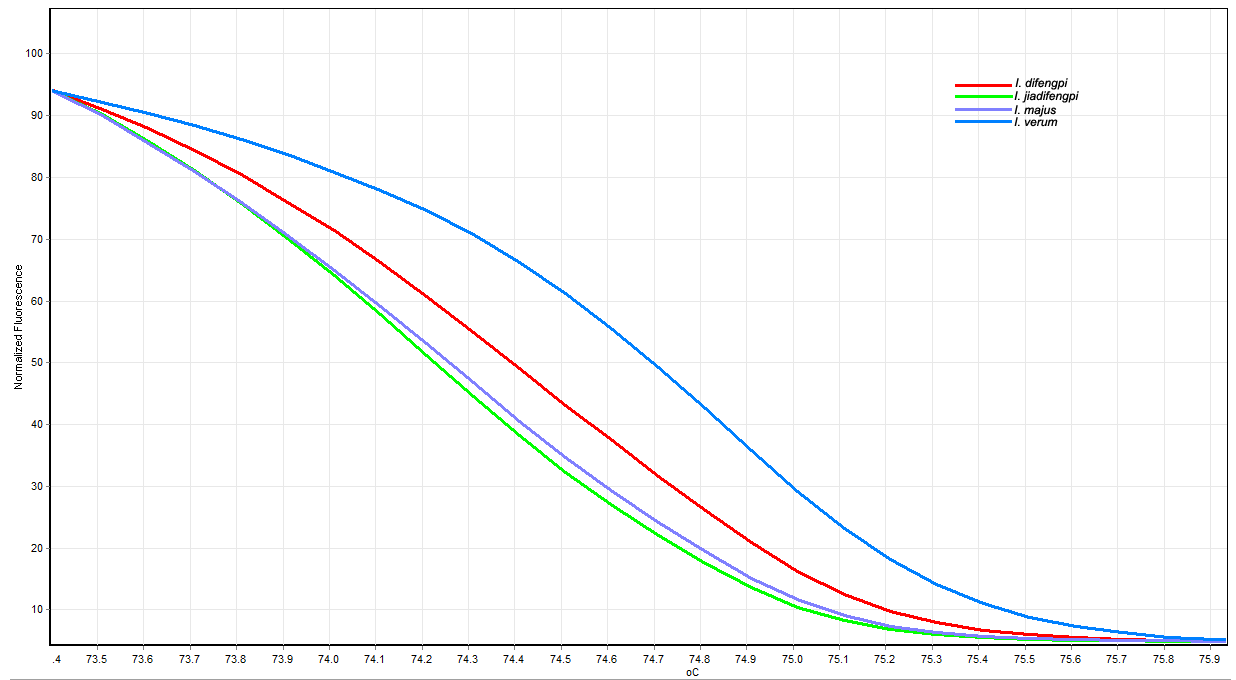


**Figure S5. Melting curves generated by primers I.DFP04_F/I.DFP04_R.** This primer pair cannot differentiate *I. jiadifengpi* from *I. majus*.


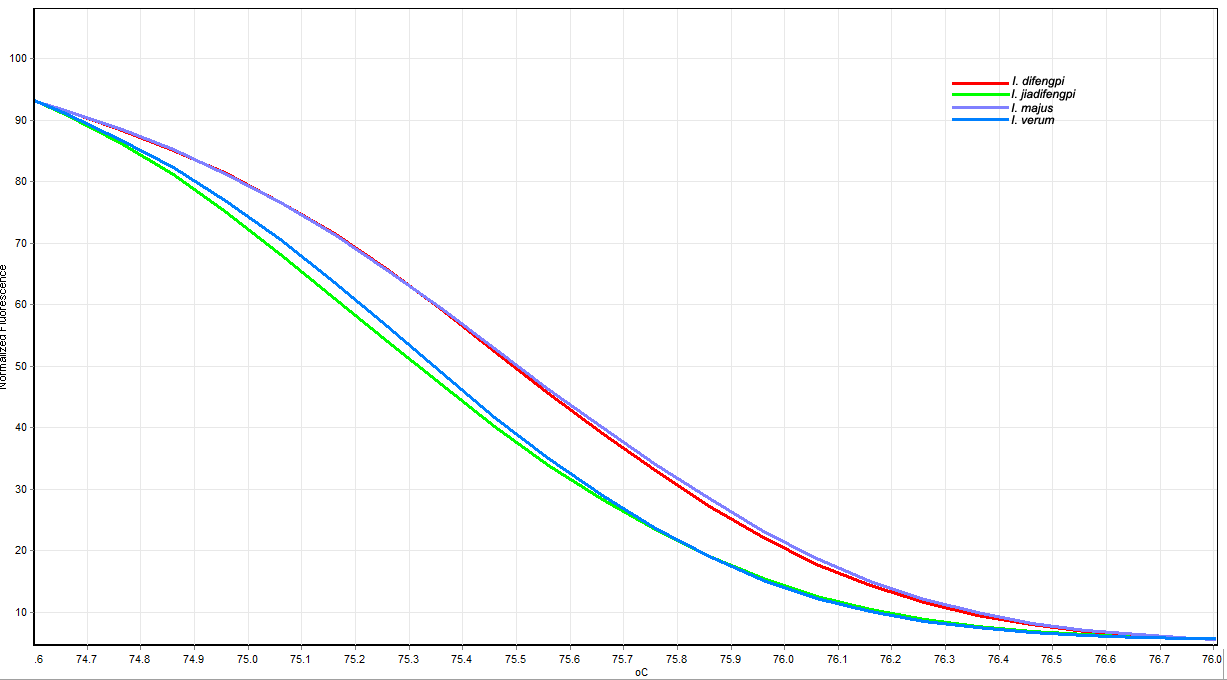


**Figure S6. Melting curves generated by primers I.DFP05_F/I.DFP05_R.** This primer pair cannot differentiate *I. jiadifengpi* from *I. verum*, and *I. difengpi* from *I. majus*.


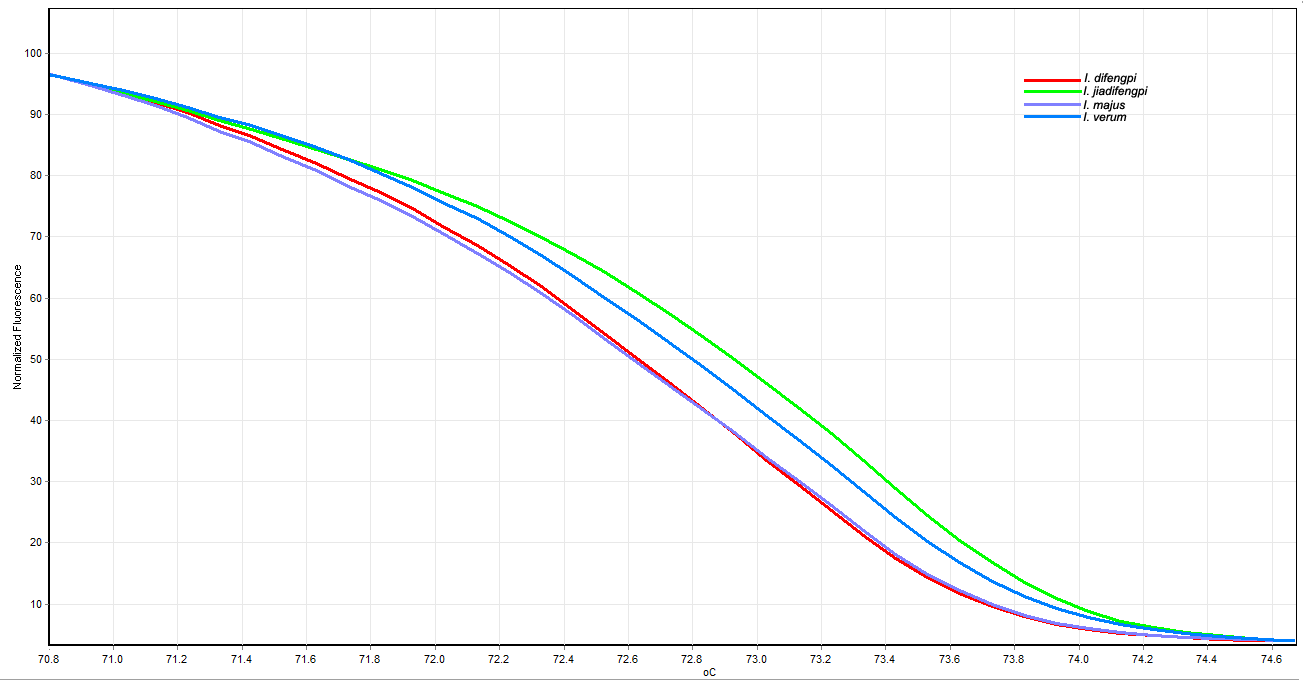


**Figure S7. Melting curves generated by primers I.DFP08_F/I.DFP08_R.** This primer pair cannot differentiate *I. jiadifengpi* from *I. verum*, and *I. difengpi* from *I. majus*.


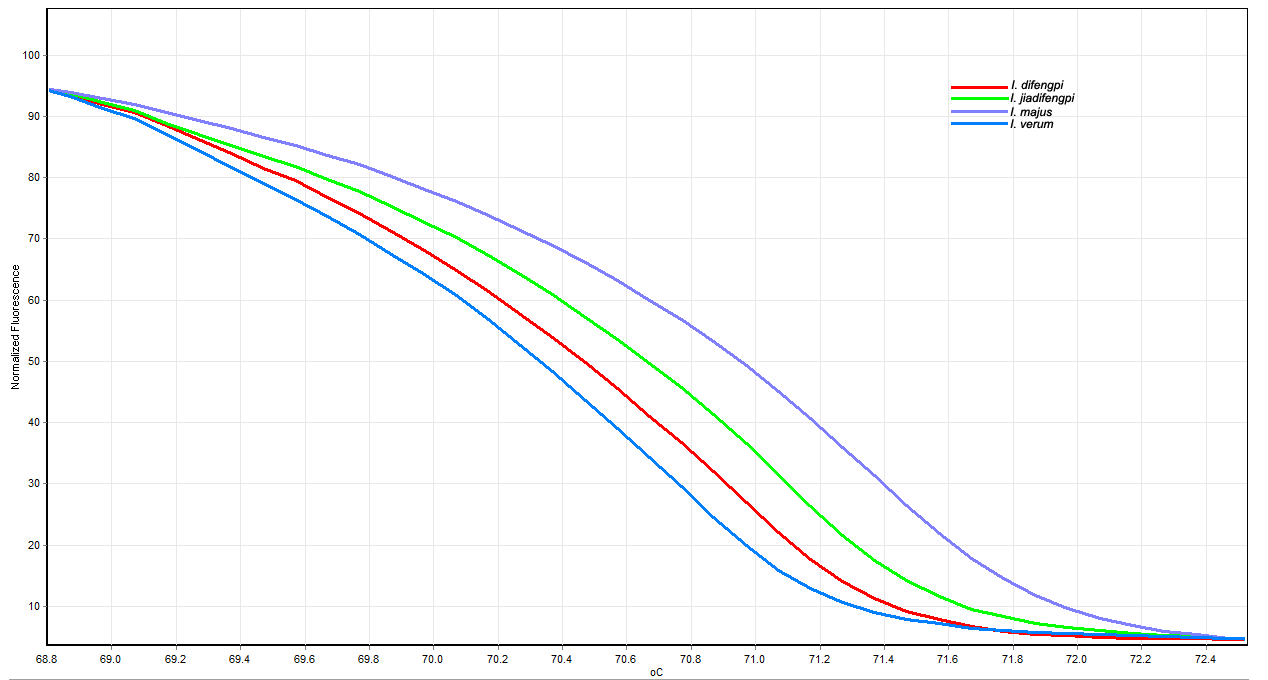


**Figure S8. Melting curves generated by primers I.DFP09_F/I.DFP09_R.** This primer pair cannot differentiate *I. difengpi* from *I. verum*.


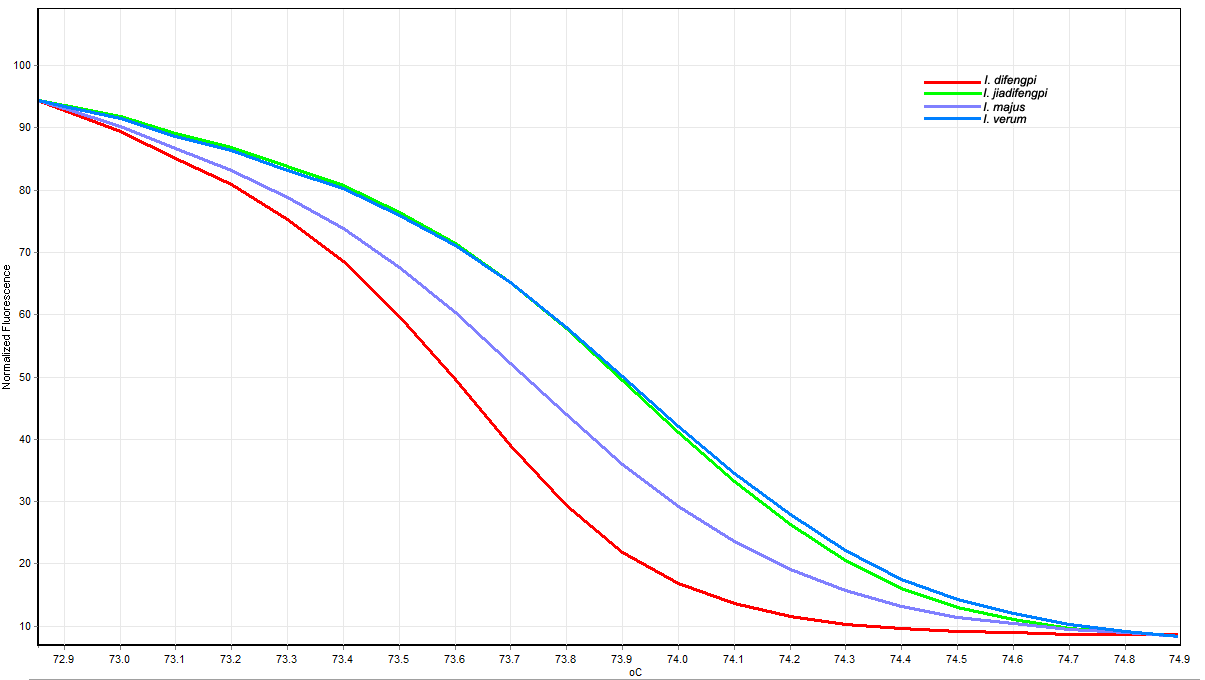


**Figure S9. Melting curves generated by primers I.DFP10_F/I.DFP10_R.** This primer pair cannot differentiate *I. jiadifengpi* from *I. verum*.


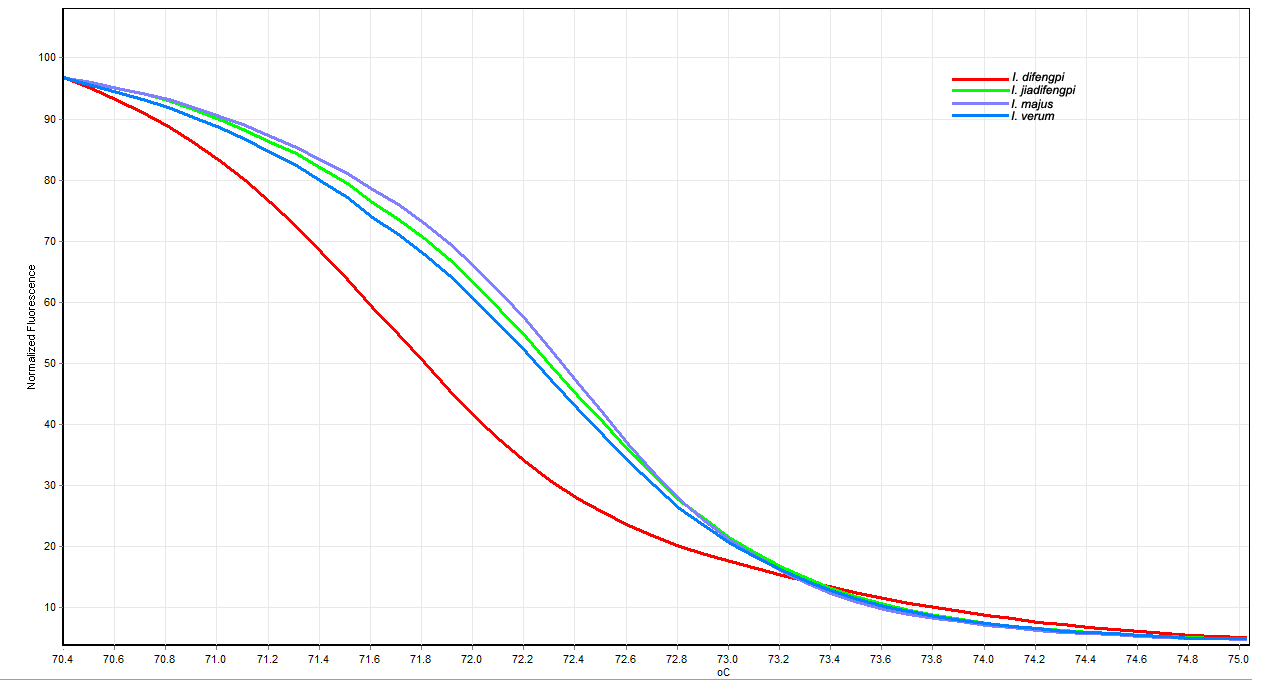


**Figure S10. Melting curves generated by primers I.DFP11_F/I.DFP11_R.** This primer pair cannot differentiate *I. jiadifengpi*, *I. majus* and *I. verum* from each other.


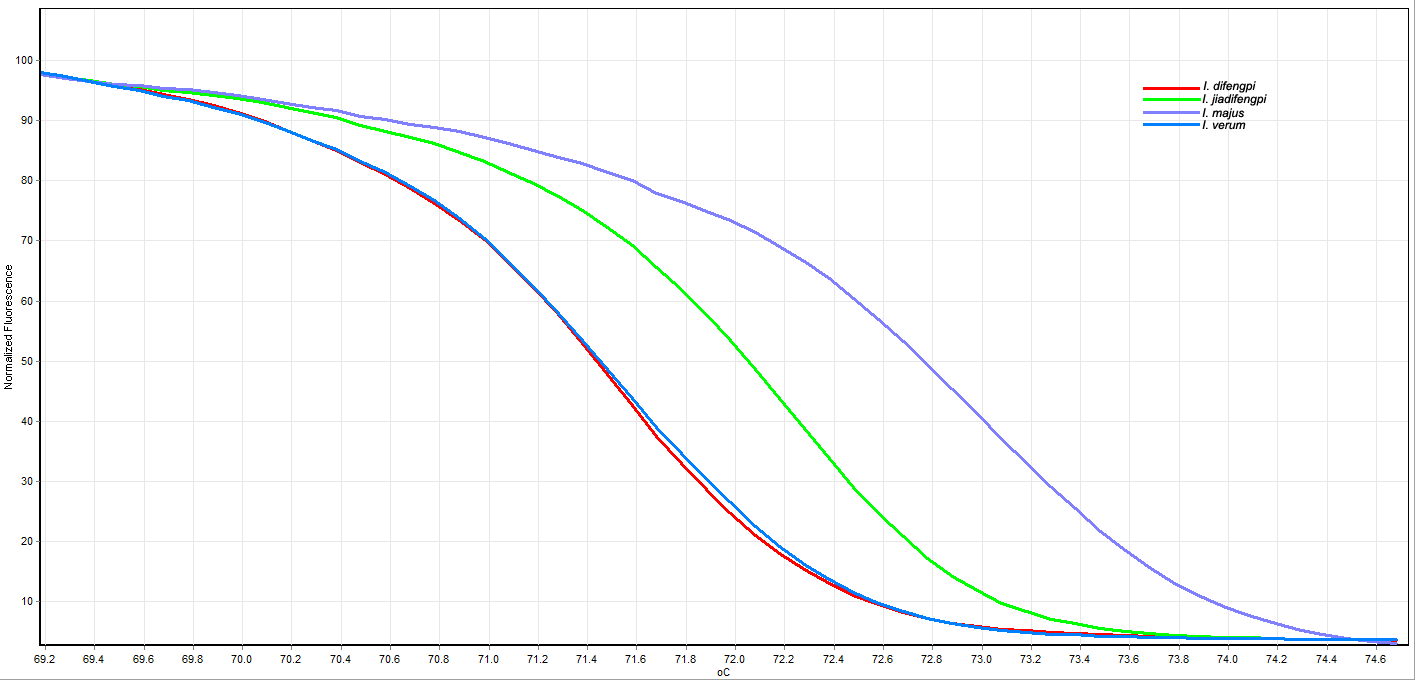


**Figure S11. Melting curves generated by primers I.DFP12_F/I.DFP12_R.** This primer pair cannot differentiate *I. difengpi* from *I. verum*.


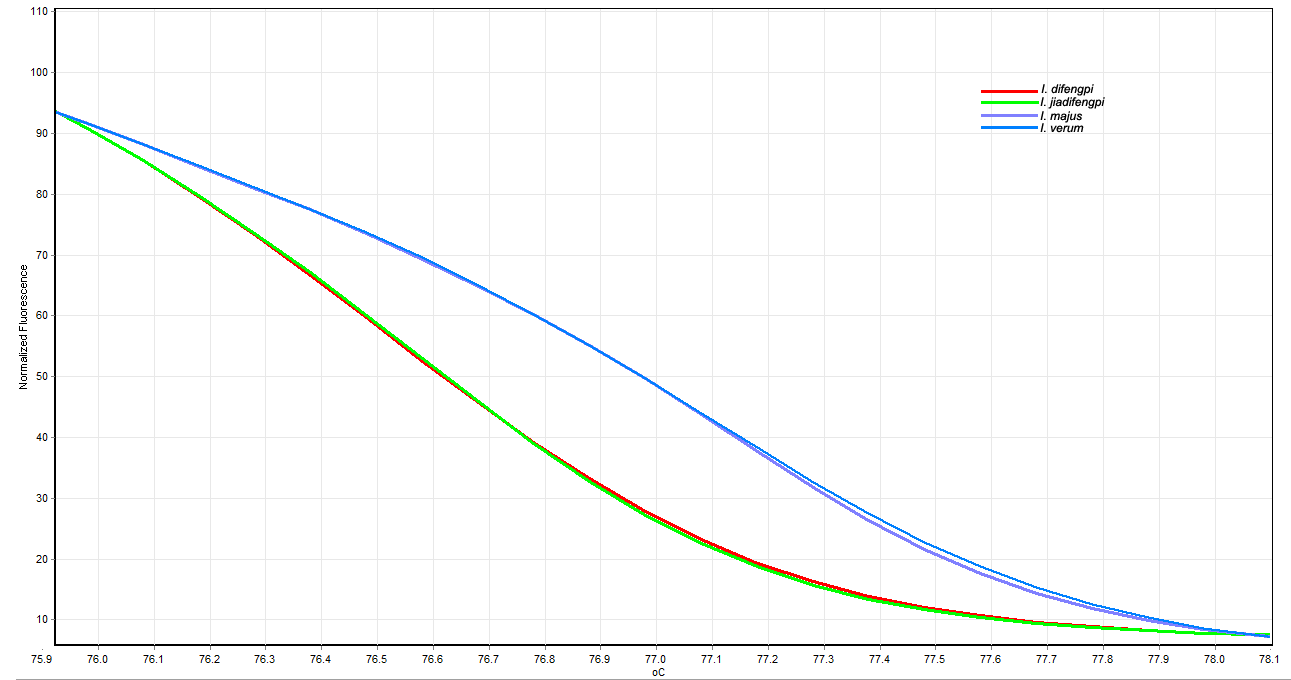


**Figure S12. Melting curves generated by primers I.DFP13_F/I.DFP13_R.** This primer pair cannot differentiate *I. difengpi* from *I. jiadifengpi*, and *I. majus* from *I. verum*.


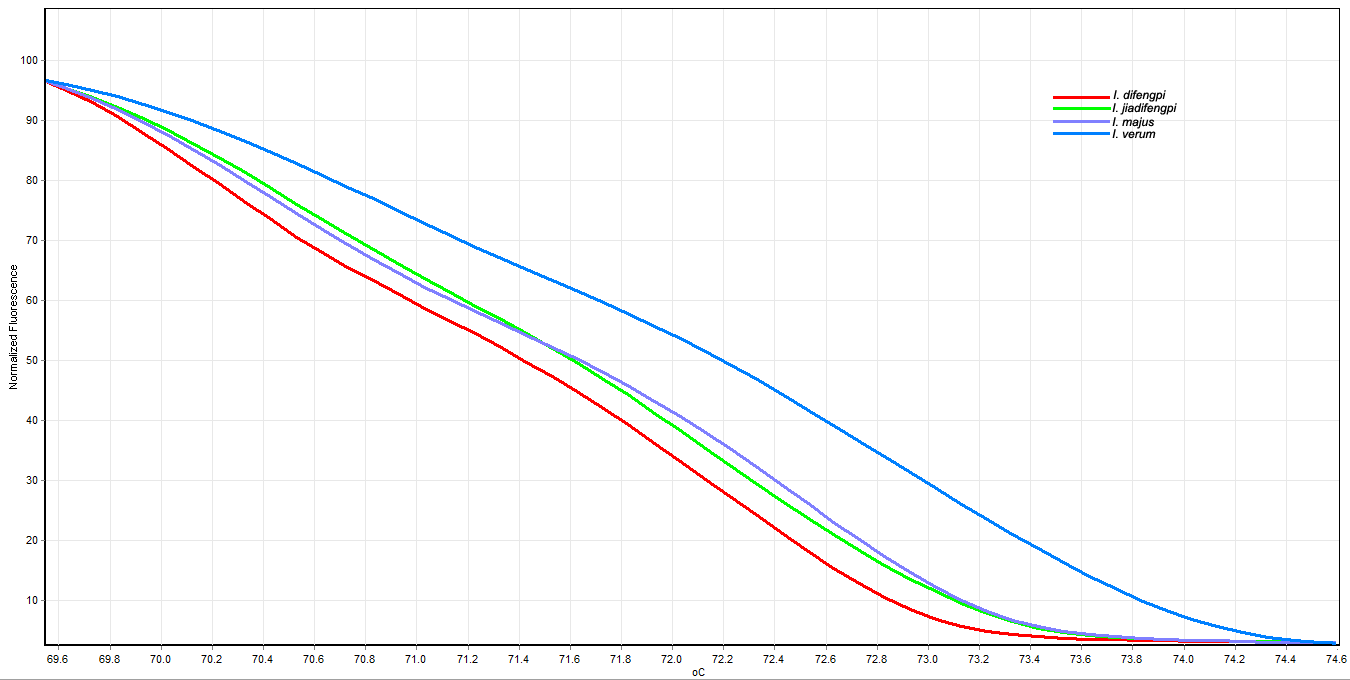


**Figure S13. Melting curves generated by primers I.DFP15_F/I.DFP15_R.** This primer pair cannot differentiate *I. difengpi*, *I. jiadifengpi* and *I. majus* from each other.


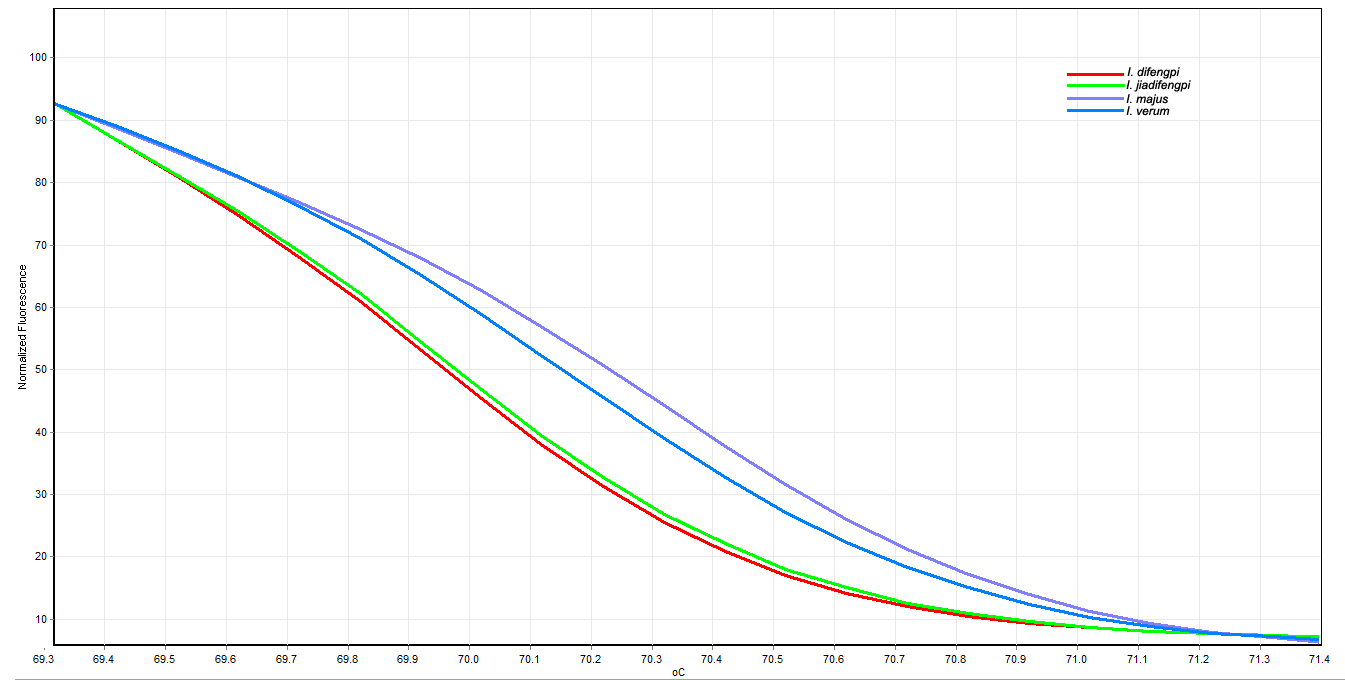


**Figure S14. Melting curves generated by primers I.DFP16_F/I.DFP16_R.** This primer pair cannot differentiate *I. difengpi* from *I. jiadifengpi*, and *I. majus* from *I. verum*.


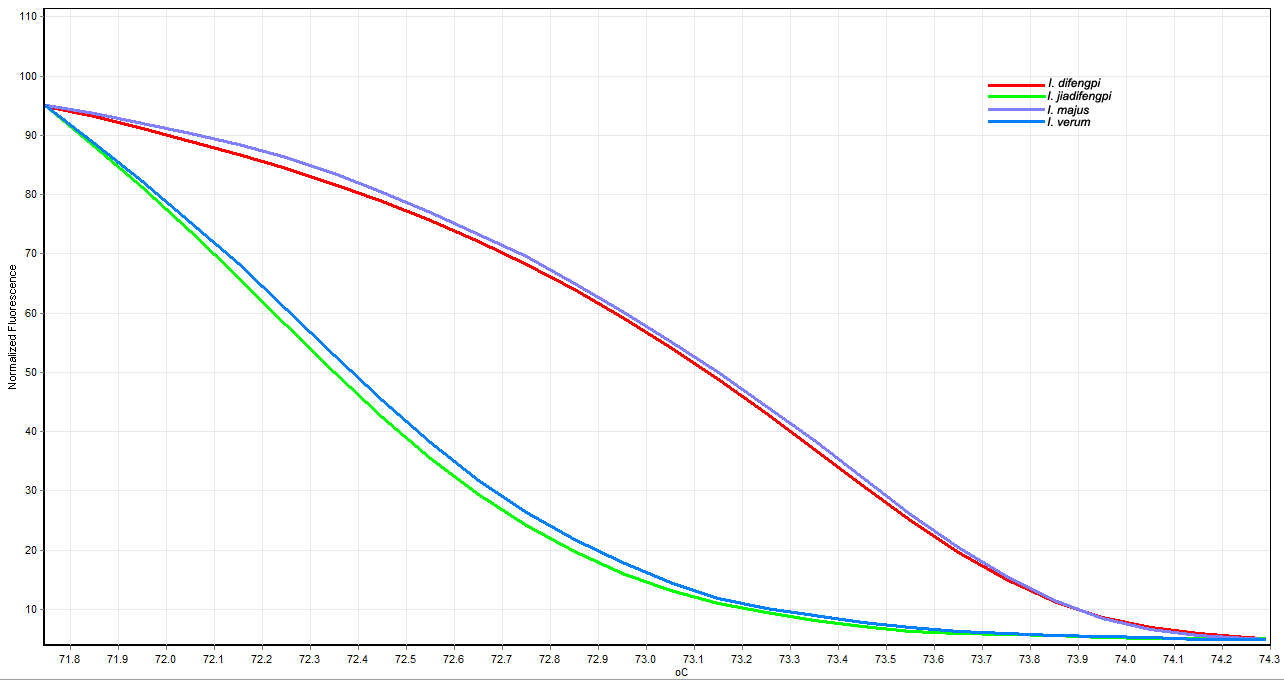


**Figure S15. Melting curves generated by primers I.DFP17_F/I.DFP17_R.** This primer pair cannot differentiate *I. difengpi* from *I. majus*, and *I. jiadifengpi* from *I. verum*.


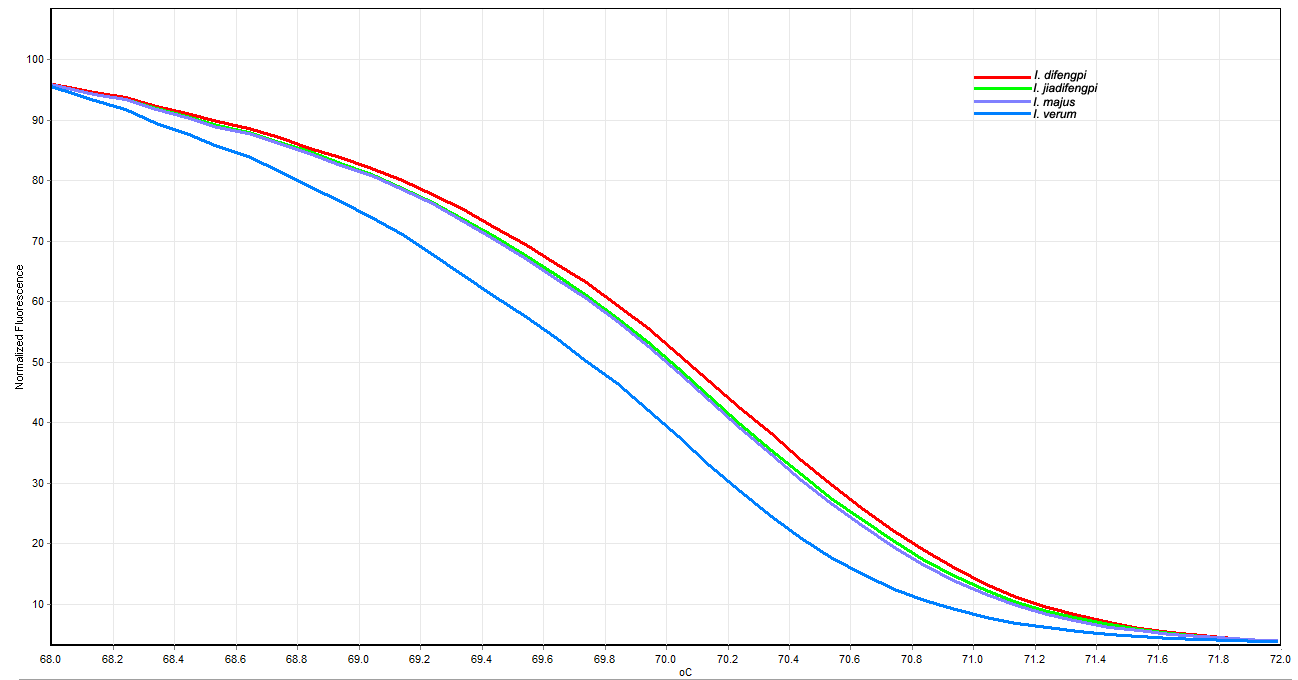


**Figure S16. Melting curves generated by primers I.DFP19_F/I.DFP19_R.** This primer pair cannot differentiate *I. difengpi*, *I. majus* and *I. jiadifengpi* from each other.


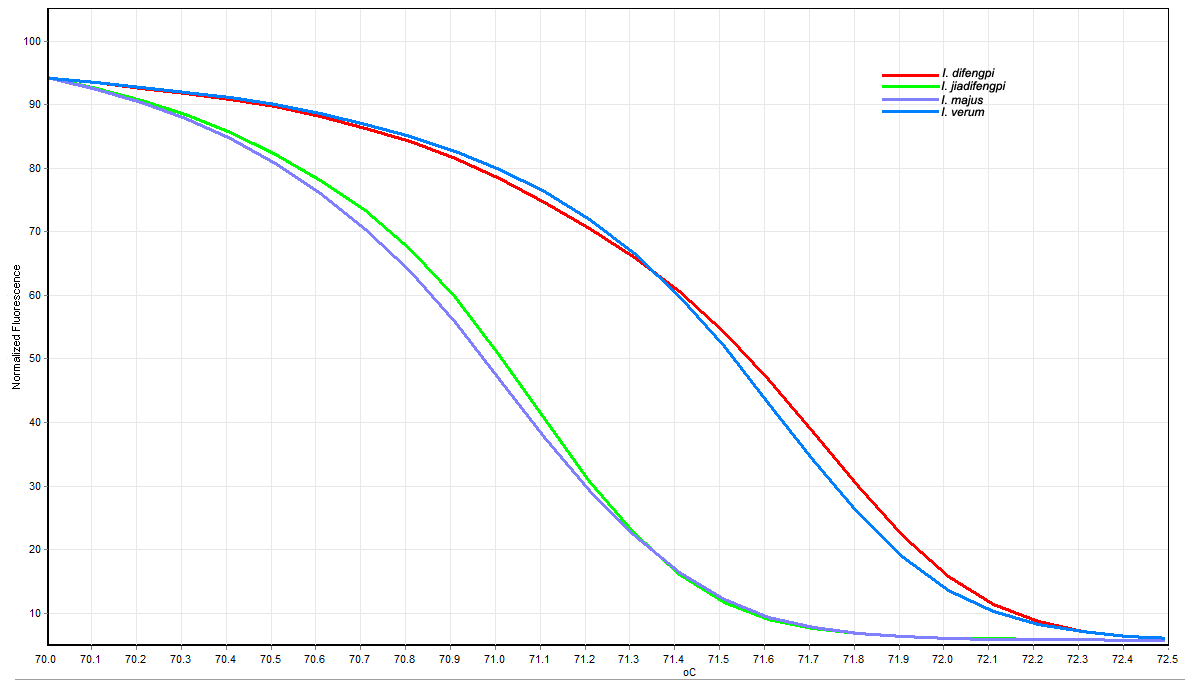


**Figure S17. Melting curves generated by primers I.DFP20_F/I.DFP20_R.** This primer pair cannot differentiate *I. jiadifengpi* from *I. majus*, and *I. difengpi* from *I. verum*.


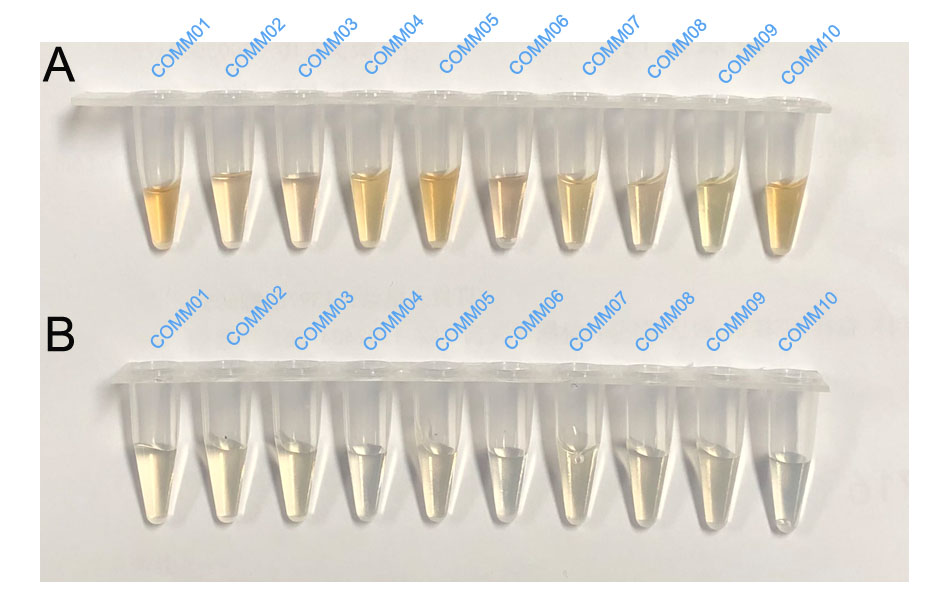


**Figure S18. DNA characteristic of 10 commercial Difengpi products. A. DNA was extracted directly using Plant DNA extraction kit without washing pre-treatment. B. DNA was extracted using Plant DNA extraction kit with washing pre-treatment.**

**Table S3. DNA concentration and purity ratios of 10 commercial Difengpi products**

| Extracted method | Sample name | Concentration  (ng/uL) | A260/A280 | A260/A230 |
| --- | --- | --- | --- | --- |
| DNA was extracted directly using Plant DNA extraction kit without washing pre-treatment. | COMM01 | 677.2 | 1.35 | 0.25 |
|  | COMM02 | 547.5 | 1.39 | 0.30 |
|  | COMM03 | 1453.8 | 1.27 | 0.24 |
|  | COMM04 | 432.1 | 1.19 | 0.25 |
|  | COMM05 | 411.7 | 1.40 | 0.44 |
|  | COMM06 | 370.4 | 1.51 | 0.56 |
|  | COMM07 | 1009.0 | 0.79 | 0.54 |
|  | COMM08 | 236.4 | 0.91 | 0.36 |
|  | COMM09 | 479.1 | 0.73 | 0.45 |
|  | COMM10 | 492.8 | 0.72 | 0.41 |
| DNA was extracted directly using Plant DNA extraction kit with washing pre-treatment. | COMM01 | 332.2 | 1.52 | 0.63 |
|  | COMM02 | 241.2 | 1.45 | 0.56 |
|  | COMM03 | 312.7 | 1.53 | 0.63 |
|  | COMM04 | 337.7 | 1.55 | 0.65 |
|  | COMM05 | 310.2 | 1.68 | 0.80 |
|  | COMM06 | 315.7 | 1.53 | 0.64 |
|  | COMM07 | 294.6 | 1.66 | 0.77 |
|  | COMM08 | 324.6 | 1.56 | 0.66 |
|  | COMM09 | 363.4 | 1.57 | 0.68 |
|  | COMM10 | 238.2 | 1.67 | 0.78 |


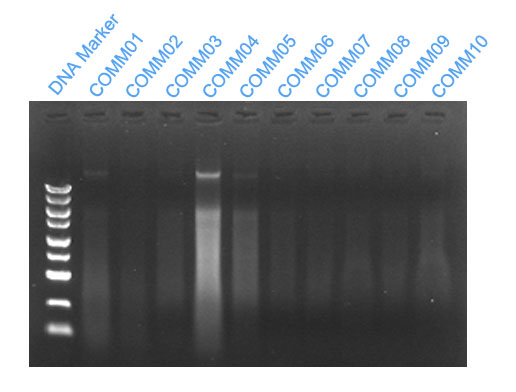


**Figure S19. Agarose gel electrophoresis of DNA extracted from 10 commercial Difengpi products.**

**Table S4. Genotype confidence percentage of *trnL*–*trnF* between four *Illicium* species.**

| Species | *I. difengpi* | *I. jiadifengpi* | *I. majus* | *I. verum* |
| --- | --- | --- | --- | --- |
| *I. difengpi* | 100.00 | 8.43 | 49.69 | 0.00 |
| *I. jiadifengpi* | 8.43 | 100.00 | 55.83 | 0.09 |
| *I. majus* | 49.69 | 55.83 | 100.00 | 0.00 |
| *I. verum* | 0.00 | 0.09 | 0.00 | 100.00 |

**Table S5. Genotype confidence percentage of *ycf1*–*ndhF* between four *Illicium* species.**

| Species | *I. difengpi* | *I. jiadifengpi* | *I. majus* | *I. verum* |
| --- | --- | --- | --- | --- |
| *I. difengpi* | 100.00 | 29.52 | 0.00 | 0.00 |
| *I. jiadifengpi* | 29.52 | 100.00 | 0.00 | 0.24 |
| *I. majus* | 0.00 | 0.00 | 100.00 | 55.03 |
| *I. verum* | 0.00 | 0.24 | 55.03 | 100.00 |

**Table S6. Genotype confidence percentage of *rpl32*–*trnL* between four *Illicium* species.**

| Species | *I. difengpi* | *I. jiadifengpi* | *I. majus* | *I. verum* |
| --- | --- | --- | --- | --- |
| *I. difengpi* | 100.00 | 45.10 | 0.86 | 0.00 |
| *I. jiadifengpi* | 45.10 | 100.00 | 1.21 | 0.01 |
| *I. majus* | 0.86 | 1.21 | 100.00 | 29.58 |
| *I. verum* | 0.00 | 0.01 | 29.58 | 100.00 |
